# Dissecting the Interaction Domains of SARS-CoV-2 Nucleocapsid Protein and Human RNA Helicase DDX3X and Search for Potential Inhibitors

**DOI:** 10.3390/ijms27020672

**Published:** 2026-01-09

**Authors:** Camilla Lodola, Maria Michela Pallotta, Fabrizio Manetti, Paolo Governa, Emmanuele Crespan, Giovanni Maga, Massimiliano Secchi

**Affiliations:** 1 Institute of Molecular Genetics IGM-CNR ‘Luigi Luca Cavalli-Sforza’, Via Abbiategrasso 207, I-27100 Pavia, Italy; camilla.lodola@igm.cnr.it (C.L.); mariamichela.pallotta@igm.cnr.it (M.M.P.); emmanuele.crespan@igm.cnr.it (E.C.);; 2 Department of Biotechnology, Chemistry and Pharmacy, University of Siena, Via Aldo Moro 2, I-53100 Siena, Italypaolo.governa@unisi.it (P.G.); 3 Department of Food and Drug, University of Parma, Parco Area delle Scienze 27/A, I-43124 Parma, Italy

**Keywords:** SARS-CoV-2, dead-box RNA helicase, nucleocapsid, DDX3X, antiviral drugs

## Abstract

The SARS-CoV-2 nucleocapsid protein (Np) plays multifunctional roles in the viral life cycle. By interacting with host cellular proteins, Np regulates viral RNA transcription, replication, and immune evasion. It controls genome packaging and counteracts host RNA interference mediated antiviral responses through its RNA binding activity. Previous studies revealed a physical interaction between Np and DDX3X, a human DEAD-box RNA helicase that facilitates the replication of several viruses. This interaction enhances Np affinity for double-stranded RNA and inhibits DDX3X helicase activity. Since Np-RNA binding activity promotes ribonucleoprotein complex formation, targeting this interaction is a promising antiviral strategy. We generated truncated protein variants to define interaction regions between Np and DDX3X. Using AlphaFold modelling, we identified RecA2 as the key DDX3X domain involved in Np binding. Finally, to disrupt Np-RNA complex formation, we screened a small molecule library of putative binders of Np N-terminal region and identified two candidate inhibitors for further development.

## 1. Introduction

The emergence of SARS-CoV-2, the virus responsible for the COVID-19 pandemic, has prompted extensive research to understand its molecular biology and host cells interaction. SARS-CoV-2, as a positive-sense RNA virus, relies on a complex network of protein–protein and protein–RNA interactions to hijack host cell machinery to optimize viral replication and immune evasion [1].

Among the viral proteins, Np plays a central role in the viral life cycle. Besides its structural function in encapsidation of the viral genome into ribonucleoprotein complex (RNP), Np controls the host cell processes that determine immune response and viral replication [2,3]. Np facilitates compaction of the ~30 kb genomic RNA (gRNA) through RNA binding and liquid–liquid phase separation (LLPS) [4,5,6]. Np is composed of two folded domains: the N-terminal domain (NTD), which mediates RNA binding and viral genome packaging, and the C-terminal domain (CTD), which promotes dimerization and contributes to RNA binding [7]. These domains are flanked by intrinsically disordered regions (IDRs), including the N-arm, the Ser-Arg–rich central linker region (LKR), and the C-tail, which promote liquid–liquid phase separation and support viral RNP assembly [8,9]. In addition to its structural role, Np is involved in viral RNA replication, subgenomic RNA transcription, and modulation of host innate immune responses by interacting with multiple host proteins [10].

Notably, we have previously demonstrated that Np physically interacts with the human DEAD-box RNA helicase DDX3X, enhancing its double-stranded RNA (dsRNA) binding affinity and potentially altering the stress granule machinery [11,12]. DDX3X is a multifunctional DEAD-box RNA helicase involved in a wide range of biological processes, including RNA metabolism, cell growth, and innate immunity. It is a key player in RNA transcription, mRNA export, translation, and in the resolution of RNA:DNA hybrids. The ATPase and helicase functions of DDX3X allow it to remodel RNA, while the N-terminus and C-terminus domains contribute to its ability to engage in numerous protein–protein interactions [13,14]. In innate immunity, DDX3X functions as an adaptor in the RIG-I–like receptor (RLR) pathway, binding viral dsRNA and recruiting interferon-beta promoter stimulator 1 (IPS-1) to induce type I interferon β production [15,16,17]. It is also ubiquitinated by TRIM25 to enhance immune stimulation [18]. Significantly, DDX3X is a required factor in the replication of several viruses, such as HIV-1, hepatitis C virus (HCV), and SARS-CoV-2, and it is a promising target for antiviral therapy [19,20]. Consistently, several small-molecule inhibitors targeting DDX3X have been identified and characterized. The most extensively studied compound, RK-33, inhibits the ATPase/helicase activity of DDX3X and has demonstrated both antitumor and broad-spectrum antiviral activity, including reported effects on SARS-CoV-2 replication. Additional compounds developed more recently further support the therapeutic potential of targeting DDX3X-mediated pathways in viral infections [21].

In addition to DDX3X, other RNA helicases such as DDX1, DDX5, DDX6, and DDX21 have been found to be engaged in the recognition of viral dsRNA and in the induction of antiviral responses [10,22]. These findings suggest that SARS-CoV-2 may exploit host helicases for suppressing innate immunity and promoting viral replication. Other Np-interacting host factors include RNA-processing proteins such as RPL36, G3BP1/2, and LARP1, whose role in viral pathogenesis and immune evasion are under active investigation [23,24]. Overall, the diverse and complex interactome of SARS-CoV-2 N protein with host factors highlights the ability of the virus to manipulate multiple cellular pathways to support viral replication and immune system control. The characterization of these interactions represents an important step towards the identification of potential antiviral targets that disrupt the virus–host interface [9,25].

To better define the contact regions between Np and DDX3X, we purified the NTD and CTDs of Np, as well as several truncated forms of DDX3X. Then, we evaluated their interaction with EMSA, Pull-Down and NanoBRET assays. Finally, to explore the possibility of developing antiviral compounds that block the formation of Np-RNA complex, we tested a series of small molecules designed as putative ligands of Np NTD.

## 2. Results

### 2.1. Human DDX3X Increases the Affinity of SARS-CoV-2 Np CTD and NTD Protein for dsRNA

**Scheme 1:**
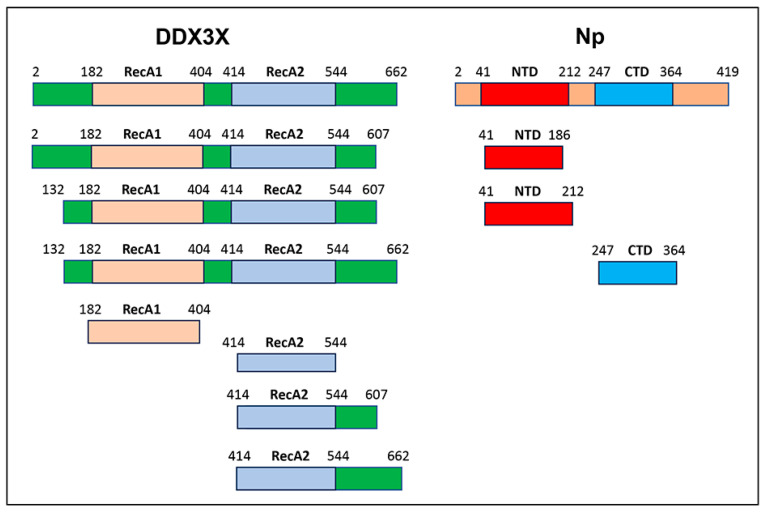
Graphical representation of the amino acid regions of the truncated DDX3X and Np domains used in the experiments.

**Figure 1:**
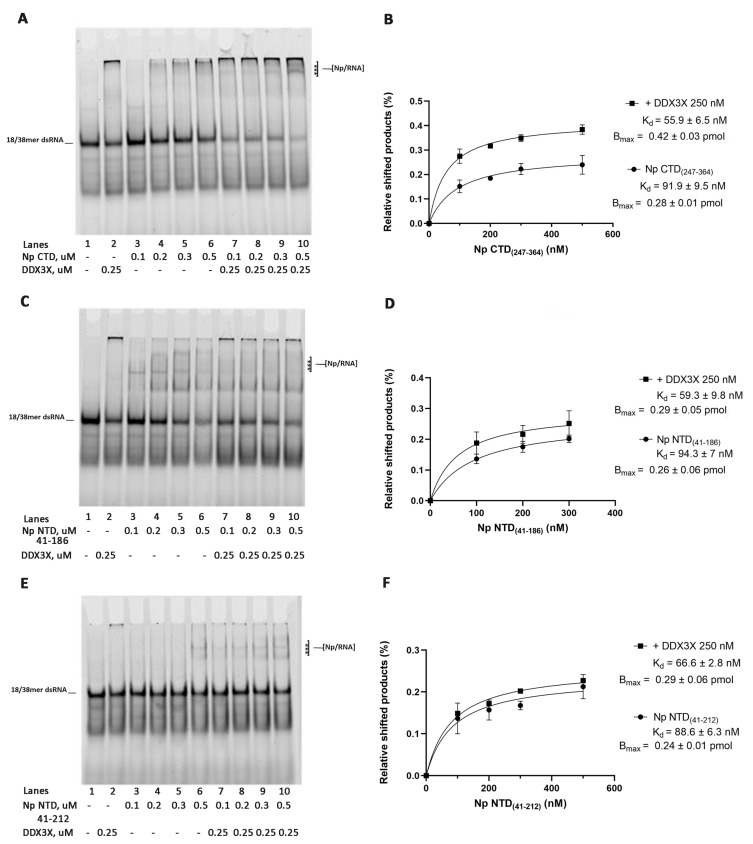
DDX3X increases the affinity of SARS-CoV-2 Np protein for dsRNA. Asterisks (***) in all panels indicate the high molecular weight Np–dsRNA complexes. (**A**) EMSA showing the titration of Np CTD_(247–364)_ in the presence of dsRNA and in the absence (lanes 3–5) or in presence (lanes 7–10) of DDX3X. Lane 1—control in the absence of proteins; Lane 2—control in the presence of DDX3X. (**B**) Np–dsRNA complex formation as a function of Np CTD concentrations in the absence (circles) or in the presence (squares) of the DDX3X. (**C**) As in (**A**), but with Np NTD_(41–186)_ replacing Np CTD. (**D**) Np–dsRNA complex formation as a function of Np NTD_(41–186)_ concentrations in the absence (circles) or in the presence (squares) of the DDX3X. (**E**) As in (**A**), but with Np NTD_(41–212)_ replacing Np CTD. (**F**) Np–dsRNA complex formation as a function of Np NTD_(41–212)_ concentrations in the absence (circles) or in the presence (squares) of the DDX3X. Curves in (**B**,**D**,**F**) were fitted with the software GraphPad Prism 9.0. and the data points represent the mean of three independent experiments ± S.E.

To investigate whether DDX3X influences the dsRNA binding affinity of the isolated NTD and CTDs of SARS-CoV-2 Np, Np NTD and Np CTD titrations were performed in the presence of 18/38mer dsRNA and in the absence or presence of a fixed amount of DDX3X and were analyzed by Electrophoretic Mobility Shift Assay (EMSA). Two NTD fragments were used, namely Np NTD_(41–186)_ and Np NTD_(41–212)_ (Scheme 1). Increasing concentrations of Np CTD_(247–364)_ and both Np NTDs alone resulted in a proportional increase in Np-RNA complexes (Figure 1A,C,E, lanes 2–5). Addition of a fixed amount of DDX3X caused an increase in the high molecular weight complexes (indicated by asterisks in Figure 1A,C,E lanes 6–9). High molecular weight bands seen in DDX3X-only controls were not considered as they represent excess of DDX3X protein that does not bind dsRNA, as confirmed by WEMSA assay (Supplementary Figure S1). Quantification of the shifted products from three independent experiments allowed estimation of apparent binding parameters, including apparent affinity (K_d_) and the maximal fraction of bound dsRNA (B_max_). Binding efficiency was estimated by the B_max_/K_d_ ratio. Np CTD alone showed a B_max_/K_d_ value of 3.1 pmol·µM^−1^, which increased to 7.5 pmol·µM^−1^ in the presence of DDX3X (Figure 1B). The Np NTD_(41–186)_ domain showed an apparent B_max_/K_d_ of 2.7 pmol·µM^−1^ and the addition of DDX3X increased the B_max_/K_d_ value up to 4.9 pmol·µM^−1^ (Figure 1D). Similarly, the Np NTD_(41–212)_ showed an apparent B_max_/K_d_ of 2.7 pmol·µM^−1^ and the addition of DDX3X resulted in an increase in the B_max_/K_d_ value to 4.4 pmol·µM^−1^ (Figure 1F). Overall, the data summarized in Table 1 indicated that the Np CTD exhibited higher dsRNA binding efficiency compared to the two Np NTDs. However, in the presence of DDX3X stimulation, the dsRNA binding affinity of Np NTD and CTDs increased.

### 2.2. Mapping the DDX3X Domain of Interaction with SARS-CoV 2 Np

**Figure 2:**
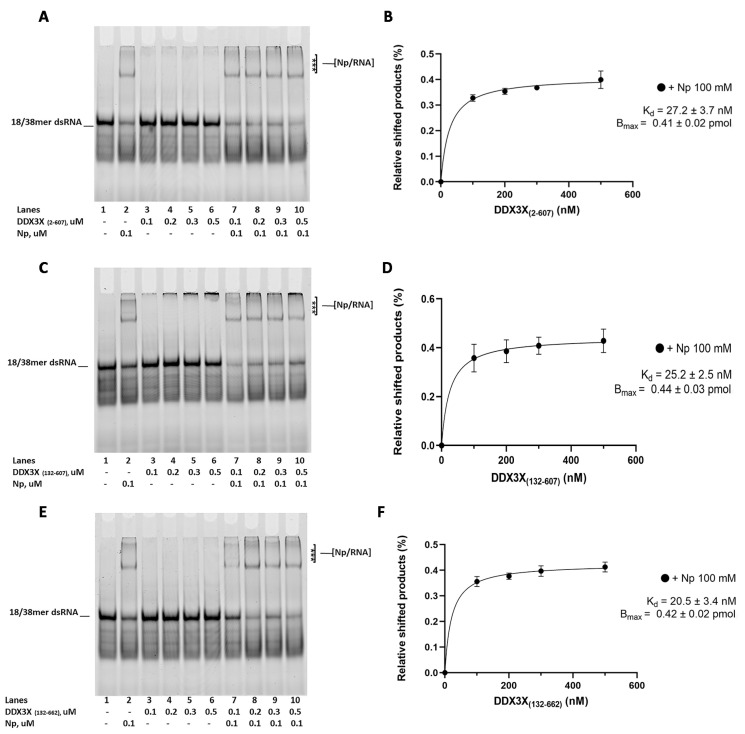
Interaction of DDX3X_(2–607)_, DDX3X_(132–607)_, DDX3X_(132–662)_ RNA helicase domains with the Np–dsRNA complex. Asterisks (***) in all panels indicate the high molecular weight Np–dsRNA complexes. (**A**) EMSA showing the titration of DDX3X_(2–607)_ in the presence of dsRNA and in the absence (lanes 3–5) or in presence (lanes 7–10) of Np. Lane 1—control on the absence of proteins; Lane 2—control in the presence of Np. (**B**) Np–dsRNA complex formation as a function of DDX3X_(2–607)_ concentrations in the presence (squares) of the Np. (**C**) As in (**A**), but with DDX3X_(132–607)_ replacing DDX3X_(2–607)_. (**D**) Np–dsRNA complex formation as a function of DDX3X_(132–607)_ concentrations in the presence (squares) of the Np. (**E**) As in (**A**), but with DDX3X_(132–662)_ replacing DDX3X_(2–607)_. (**F**) Np–dsRNA complex formation as a function of DDX3X_(132–662)_ concentrations in the presence (squares) of the Np. Curves in (**B**,**D**,**F**) were fitted with the software GraphPad Prism 9.0. and the data points represent the mean of three independent experiments ± S.E.

To identify the region of the RNA helicase DDX3X responsible for the functional interaction with SARS-CoV-2 Np, three different fragments of DDX3X, encompassing aa 2–607, 132–607 and 132–662 (Scheme 1), were expressed and purified from *E. coli*. Titrations of the corresponding DDX3X polypeptides were performed in the presence of 18/38mer dsRNA, either alone or in combination with a fixed amount of Np, and analyzed by EMSA. Increasing concentrations of DDX3X_(2–607)_, DDX3X_(132–607)_ and DDX3X_(132–662)_ alone did not result in any shifting of dsRNA (Figure 2A,C,E lanes 3–6), reflecting their weak or transient interaction with the RNA substrate. As expected, Np alone produced the characteristic Np-RNA complex (Figure 2A,C,E lane 2). Next, increasing concentrations of each DDX3X polypeptide were titrated in the presence of a fixed amount of Np (Figure 2A,C,E lanes 7–10). As shown in Figure 2B,D,F, addition of all three DDX3X polypeptides stimulated the Np-dsRNA complex formation in a dose-dependent manner, suggesting cooperative interaction. Quantification of the shifted bands from three independent experiments enabled the calculation of the apparent binding parameters for the DDX3X-Np-dsRNA interaction. By plotting the increase in the shifted products as a function of DDX3X concentration, K_d_ and B_max_ values were determined (Table 1). Binding efficiency of DDX3X to the Np-dsRNA was estimated by the B_max_/K_d_ ratio. DDX3X_(2–607)_ showed an apparent K_d_ of 27.2 nM and a B_max_/K_d_ of 15.1 pmol·µM^−1^. The DDX3X_(132–607)_ fragment had an apparent K_d_ of 25.2 nM and a B_max_/K_d_ value of 17.4 pmol·µM^−1^. Finally, DDX3X_(132–662)_ has an apparent K_d_ of 20.5 nM and a B_max_/K_d_ value of 20.5 pmol·µM^−1^. These data showed that the DDX3X_(132–662)_ moiety was the one with the highest binding affinity to the Np-dsRNA complex, compared to DDX3X_(2–607)_ and DDX3X_(132–607)_ fragments.

### 2.3. SARS-CoV-2 Np Physically Interacts with DDX3X

**Figure 3:**
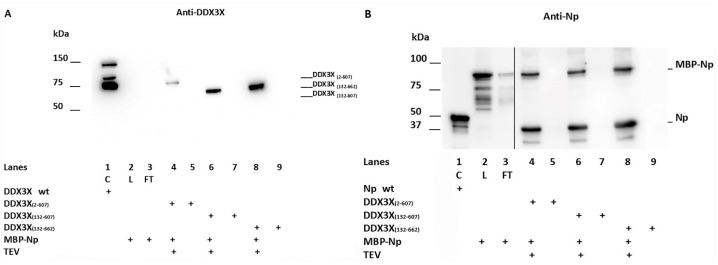
Human DDX3X_(2–607)_, DDX3X_(132–607)_ and DDX3X_(132–662)_ proteins physically interact with full-length Np. (**A**) Western blot with anti-DDX3X Abs of the fractions eluted from the MBP affinity beads with TEV protease. Lane 1—control with the recombinant MBP-DDX3X/DDX3X wt; Lane 2—loading of *E. coli* expressing recombinant MBP-tagged Np crude extract; Lane 3—flow through; Lane 4—DDX3X_(2–607)_ incubated with the beads in the presence of the MBP-tagged Np; Lane 5—DDX3X_(2–607)_ incubated with MBP tag captured on the beads in absence of Np; Lane 6—DDX3X_(132–607)_ incubated with the beads in the presence of the MBP-tagged Np; Lane 7—DDX3X_(132–607)_ incubated with MBP tag captured on the beads in absence of Np; Lane 8—DDX3X_(132–662)_ incubated with the beads in the presence of the MBP-tagged Np; Lane 9—DDX3X_(132–662)_ incubated with MBP tag captured on the beads in absence of Np. (**B**) Western blot with anti-Np Abs of the fractions eluted from the MBP affinity beads with TEV protease show in (**A**). Lane 1—control with purified recombinant 6His–Np full–length; Lane 2–9—samples loaded as in (**A**).

Based on the observations made in the previous experiments, we wanted to evaluate whether the interaction between Np and DDX3X_(2–607)_, DDX3X_(132–607)_ and DDX3X_(132–662)_ was direct or whether it was mediated by additional protein factors or RNA. The availability of all recombinant proteins enabled the assessment of their direct interaction by pull-down assays in the absence of additional factors. Recombinant maltose binding protein (MBP)-TEV-Np was bound to MBP affinity beads and incubated together with purified recombinant DDX3X_(2–607)_, DDX3X_(132–607)_ and DDX3X_(132–662)_. Then, the pulled-down material was eluted by TEV protease digestion. As revealed by Western blot with specific antibodies, all three recombinant versions of DDX3X bound Np (Figure 3A lanes 4, 6 and 8 and Figure 3B). DDX3X_(132–662)_ showed the greatest physical interaction with Np, consistent with the kinetic data shown in Figure 2 and Table 1. A mock reaction with DDX3X_(2–607)_, DDX3X_(132–607)_ and DDX3X_(132–662)_ alone, incubated with the MPB tag captured on the beads as a negative control did not result in their retention (Figure 3A lanes 5, 7 and 9).

**Figure 4:**
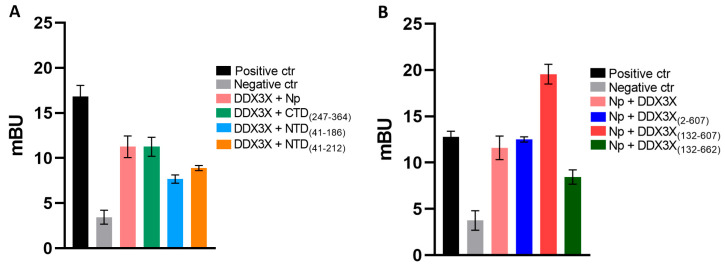
DDX3X–Np interactions with NanoBRET system expressed in Milli-BRET units (mBU). (**A**) NanoBRET of Halo–DDX3X full-length with Np wt and Np NTD and CTDs tagged with NLuc. Negative control was performed by combining Halo–DDX3X with NLuc–TGM2 (**B**) NanoBRET of NLuc–Np full-length with DDX3X wt and DDX3X truncated forms tagged with Halo. Negative control was performed by combining NLuc–Np full-length with Halo–TGM2 In both panels positive controls were performed by combining p53–Halo with NLuc–MDM2. Values are the means ± SD of two independent experiments performed in quadruplicate.

Moreover, to further validate the physical interaction between the domains of Np and truncated forms of DDX3X, we performed an additional protein–protein interaction assay using NanoBRET, a proximity-based system designed to detect intracellular protein complexes in mammalian cells. U2OS cells were transiently co-transfected with plasmids expressing the Halo-tagged DDX3X wt or its variants, along with plasmids encoding Np wt or its individual domains fused with NanoLuc (NLuc). BRET signals, indicative of close spatial proximity between two proteins, were measured 72 h post-transfection. Consistent with our EMSA results, Np full-length, as well as its NTD and CTDs showed robust interaction with DDX3X wt (Figure 4A, lanes 3–6) confirming that both domains are necessary for binding. Interestingly, all three truncated DDX3X variants were able to interact with the Np full-length protein, with the DDX3X_(132–607)_ construct displaying the highest interaction efficiency (Figure 4B, lanes 4–6). No BRET signal was observed when Halo-DDX3X was co-transfected with NLuc-TGM2, or when NLuc-Np was co-expressed with Halo-TGM2 as internal negative controls (Figure 4A,B, lanes 2). These findings were further confirmed using a Halo-TEV pull-down system. In this assay, Halo-TEV-tagged DDX3X proteins expressed in U2OS cells were captured on HaloLink resin and incubated together with the different NLuc-tagged Np domains. Luminescence signals proportional to interaction strength were measured after TEV protease treatment (Supplementary Figure S2). Collectively, these results confirm that both NTDs and CTDs of Np are important for its interaction with DDX3X.

### 2.4. AlphaFold 3 Model of DDX3X-Np Interaction

**Figure 5:**
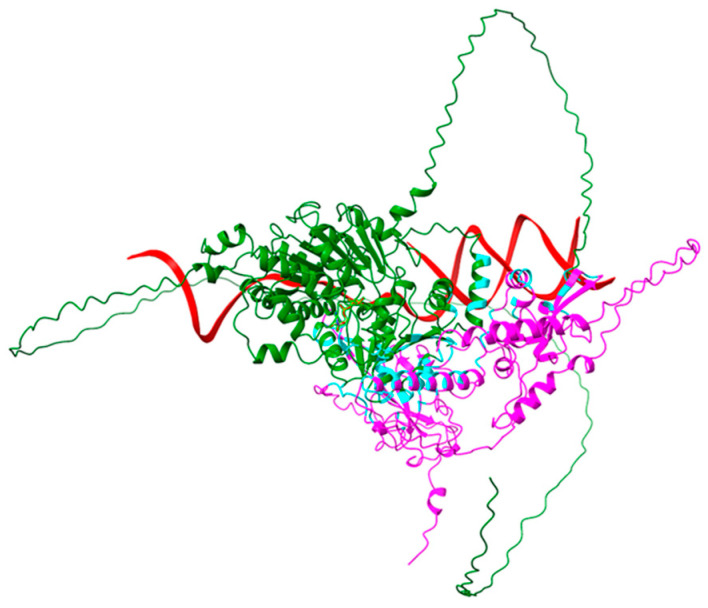
Graphical representation of the modelled three-dimensional complex between DDX3X (green), Np (magenta), 18/38mer dsRNA (red), and ATP, predicted by AlphaFold 3. The structure was analyzed with ChimeraX 1.9 to highlight amino acids (light blue) involved in interactions between Np and DDX3X.

**Figure 6:**
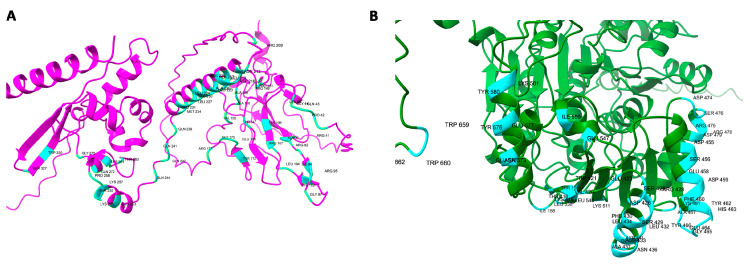
Detailed view of the interactions between DDX3X (green) and Np (magenta) analyzed with ChimeraX 1.9. (**A**) Np amino acids involved in interactions with DDX3X are shown in light blue. (**B**) DDX3X amino acids involved in interactions with Np are shown in light blue.

Next, to better define the portion of DDX3X responsible for the binding with the Np protein, we generated an AlphaFold 3 molecular model of DDX3X-Np interactions. The model included DDX3X and Np in complex with a 18/38mer dsRNA (as used in our EMSAs) and an ATP molecule. Even though the predicted complex yielded low to moderate pTM (0.52) and iPTM (0.49) scores, it was highly reliable (pLDDT > 90) or confident (90 > pLDDT > 70) for the structured core regions of both proteins, with the only exception for their unstructured N and C termini (pLDDT < 50). Moreover, as expected, the Np disordered LKR from amino acids 215 to 236 was also built with low prediction confidence and modelled as an α-helix (70 > pLDDT > 50) [26,27] (Supplementary Figure S3). The addition of dsRNA and the ATP molecule was necessary to create a more realistic model as the predicted structure with DDX3X and Np alone showed significantly lower confidence with an overall pTM score of 0.38 and iPTM of 0.24 (Supplementary Figure S4). The modelled complex was analyzed with the software ChimeraX 1.9 to identify key residues involved in the DDX3X-Np interactions (Figure 5). Remarkably, the model showed that the amino acids of Np involved in the binding with DDX3X are located within the NTD (aa 41–45, 90–97, 102–109, 149–156, 172–177) and CTD (aa 253–258, 261–263, 272–275, 327–330) domains (Figure 6A) of the viral protein that we used in our EMSA and NanoBRET/pull-down assays. However, additional residues within the disordered LKR (aa 213–234) of Np appeared to contribute to the interactions. Interestingly, the AlphaFold model suggested that DDX3X interacts with the Np domains mainly with amino acids located within its RecA2 domain (aa 416–441, 458–467, 474–479, 538–540, 572–581) and possibly in its C-terminal tail (aa 659–662) (Figure 6B). Moreover, superposition of our AlphaFold structure of DDX3X with the published crystal structure of DDX3X (PDB ID: 2I4I) [28] showed a remarkable structural similarity, with an overall RMSD value of 0.736 Å calculated on heavy atoms. In further detail, the RecA1 domains were almost perfectly superposed, while the RecA2 domains were rotated by about 176 degrees with a translation of 28.2 Å (Supplementary Figure S5). The presence of Np in our modelled complex is probably responsible for the different spatial arrangement of the RecA2 domain of DDX3X, which underwent a roto-translation necessary to form important interactions with both the NTD and CTDs of the viral protein.

### 2.5. DDX3X RecA2 Domain Interact with Np

**Figure 7:**
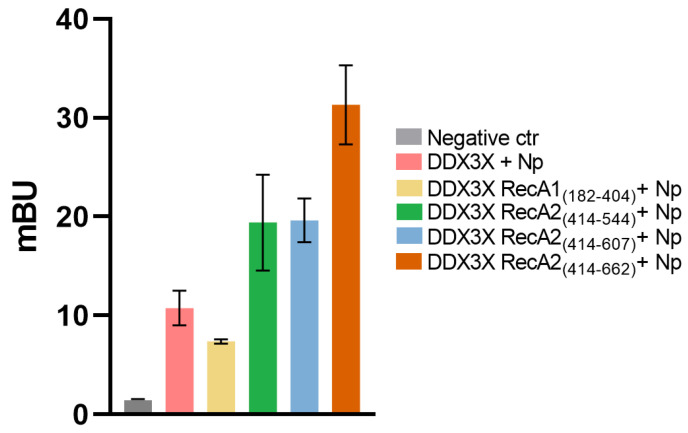
NanoBRET interaction of DDX3X RecA domains-with Np full–length expressed in Milli-BRET units (mBU). Binding data were obtained using Halo–DDX3X full-length or Halo–DDX3X RecA1 and RecA2 domains with NLuc–Np full-length. Negative control was performed by combining Halo–TGM2 with NLuc–Np full-length. Values are the means ± SD of three independent experiments performed in quadruplicate.

To better characterize the molecular interaction between DDX3X and Np, we cloned the RecA1_(182–404)_ and the RecA2_(414–544)_ domains of DDX3X in the HaloTag plasmids necessary for the expression and protein/protein interaction assays in mammalian cells. Since the AlphaFold 3 model identified DDX3X residues (572–581) just outside the RecA2 domain as potentially involved in binding, we also generated and cloned the extended fragments, RecA2_(414–607)_ and RecA2_(414–662)_, into the same HaloTag plasmid (Scheme 1). These new truncated forms of DDX3X were co-transfected with plasmid expressing the NLuc tagged versions of Np full-length in U2OS cells and BRET signals were analyzed for their interaction. Experimental results in live cells showed that DDX3X RecA1 weakly interacted with Np, while all the RecA2 constructs exhibited strong binding to the viral protein. Interestingly, the DDX3X RecA2 fragment (414–662) exhibited the highest interaction confirming the in silico prediction that also identified some DDX3X binding residues in the C-terminal sequence (Figure 7). This result was further confirmed using the Halo-TEV mammalian pull-down system (Supplementary Figure S6).

### 2.6. Search for Small Molecules Inhibitors of SARS-CoV-2 Np RNA Binding

**Figure 8:**
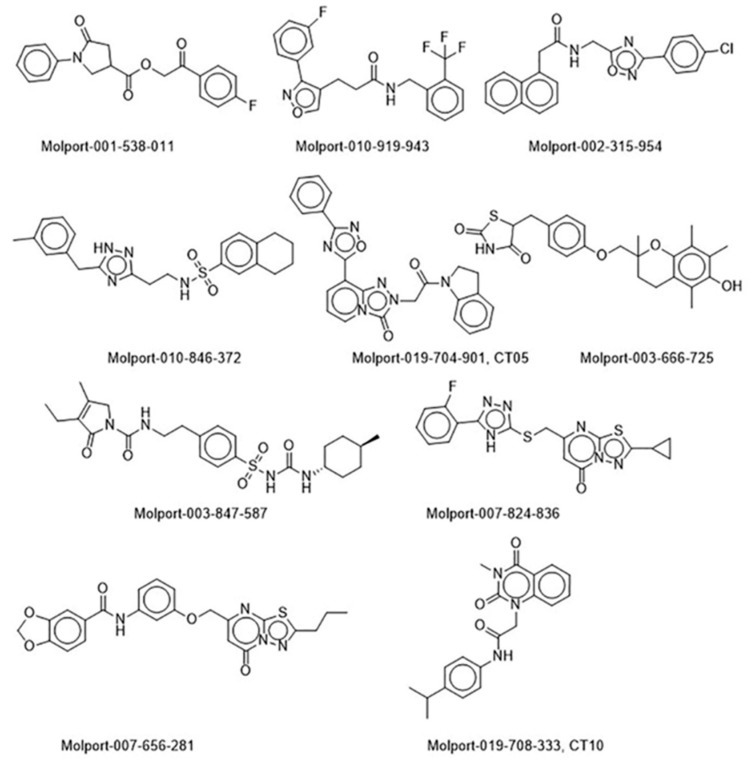
Structure of ten putative NTD ligands prioritized by virtual screening of commercially available compounds.

Np interaction with human DDX3X enhances its dsRNA binding activity which is essential in the viral replication cycle. As a consequence, the disruption of the Np-RNA interactions through small molecule inhibitors can lead to the inhibition of viral replication. In this context, molecular modelling techniques were applied to identify novel putative binders of the NTD of Np, in the attempt to affect or abrogate the interactions between Np and RNA. For this purpose, the NCI, DrugBank, and MolPort databases of commercially available compounds were filtered based on the Lipinski’s rule of five and then virtually screened using the docking software Glide on fifteen three-dimensional structures of the Np NTD (determined by experimental techniques, such as NMR or X-ray crystallography, both in the apo forms or complexed with RNA fragments). As a result, a small library of ten putative Np ligands (Figure 8) was prioritized either by choosing top-scored compounds of each docking simulation or compounds identified in more than one simulation.

**Figure 9:**
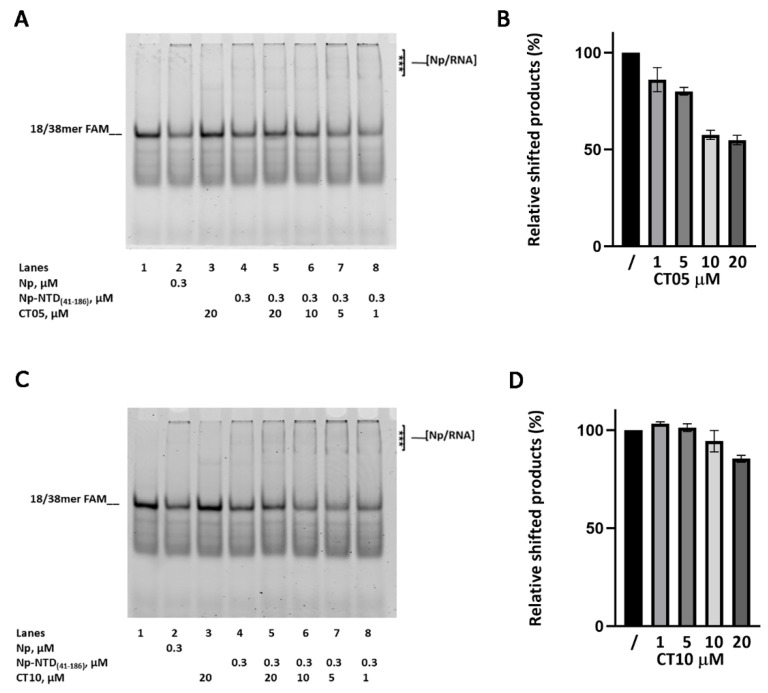
CT05 and CT10 inhibition of Np NTD_(41–186)_–dsRNA binding. Asterisks (***) in all panels indicate the high molecular weight NTD–dsRNA complexes. (**A**) EMSA showing the CT05 inhibitor effect on NTD–dsRNA binding (lanes 5–8). Lane 1—control in the absence of protein and inhibitor; Lane 2—control in the presence of Np alone; Lane 3—control in the presence of the inhibitor alone; Lane 4—control in the presence of Np NTD_(41–186)_ alone. (**B**) Reduction in NTD–dsRNA product in the presence of CT05 inhibitor indicated as a percentage of relative shifted product. Data points represent the average of two independent experiments ± SD; graphic bars were drawn using the software GraphPad Prism 9.0. (**C**) As in (**A**), but with CT10 replacing CT05. (**D**) As in (**B**), but with CT10 replacing CT05.

**Figure 10:**
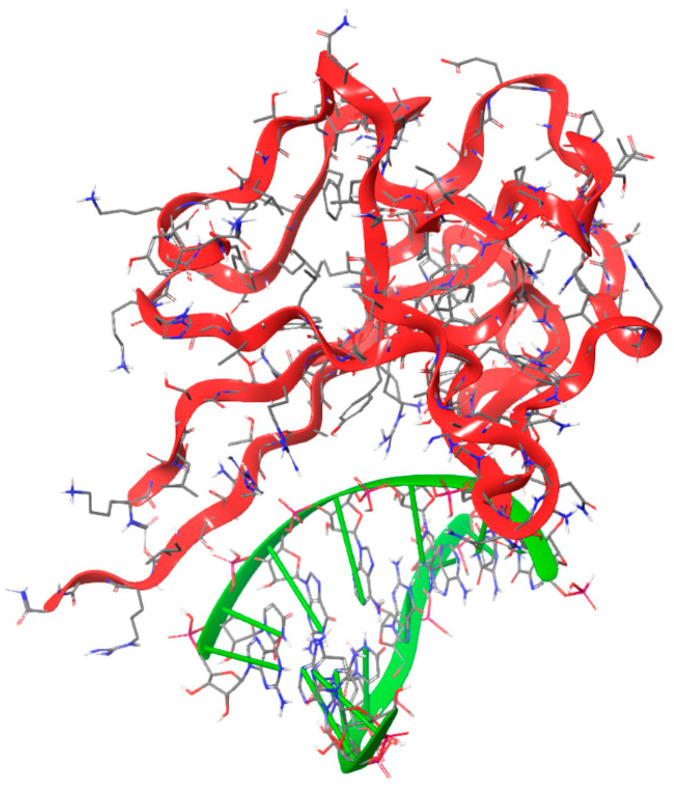
Graphical representation of the complex between the NTD of Np (stick representation and red ribbon) and a RNA sequence (stick representation and green ribbon), determined by X-ray crystallographic studies and stored within the protein data bank (7xwz).

**Figure 11:**
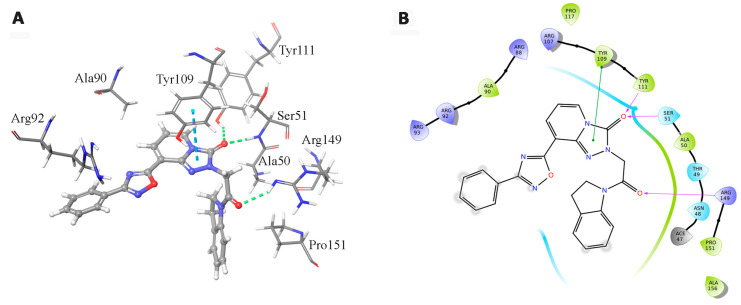
Graphical representation of the binding mode of CT05 within the RNA binding site in the Np-NTD. Only important amino acids involved in complex stabilization are displayed. (**A**) Details of the interactions between the ligand and Np (hydrogen bonds are represented by green dashed lines, π-π interactions are represented by cyan dashed lines). (**B**) Bidimensional representations of the interactions.

**Figure 12:**
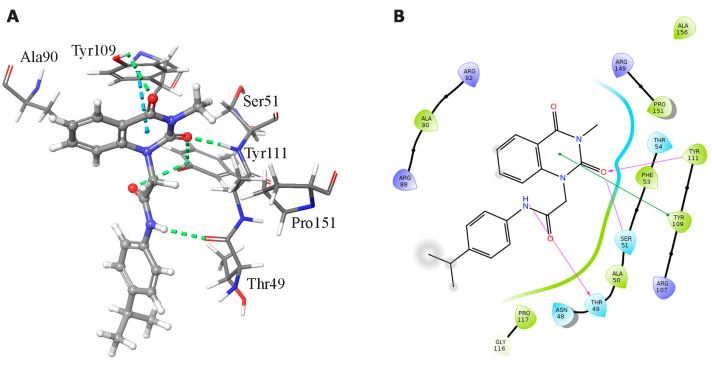
Graphical representation of the binding mode of CT10 within the RNA binding site in the Np-NTD. The ligand is accommodated within the same portion of the RNA binding site where CT05 was also located. The most important interactions are common to both ligands. Only important amino acids involved in complex stabilization are displayed. (**A**) Details of the interactions between the ligand and Np (hydrogen bonds are represented by green dashed lines, π-π interactions are represented by cyan dashed lines). (**B**) Bidimensional representations of the interactions.

Among them, CT05 and CT10 showed activity in EMSA (Figure 9). In particular, CT05 reduced by almost 50% the Np NTD_(41–186)_-dsRNA binding at a concentration of 10 µM, while CT10 was effective at 20 µM. Both compounds interacted with the RNA binding site on NTD, thus representing promising scaffolds for further optimization. In the case of the docked complex built with the Np structure stored in 7xwz (taken as the representative example of all the NP structures studied, Figure 10), the endocyclic carbonyl oxygen atom of CT05 served as a hydrogen bond acceptor for both the phenolic hydroxyl group of Tyr111 and the backbone NH moiety of Ser51 (Figure 11). Another hydrogen bond was found between the exocyclic carbonyl oxygen atom of the hit compound and the terminal guanidino moiety of Arg149. The complex was further stabilized by a π-π parallel stacked interaction between the aromatic portion of Tyr109 and the central bicyclic core of the ligand. Hydrophobic contacts involved the polymethylene portion of the Arg92 side chain, Ala50 and Ala90 methyl groups, as well as the cyclic portion of Pro151. CT10 occupied the same region of the RNA binding site and showed similar interactions (Figure 12). In fact, the endocyclic oxygen atom showed a bifurcated hydrogen bond with both the phenolic hydroxyl group of Tyr111 and the backbone NH moiety of Ser51. Moreover, a π-π parallel stacked interaction between the aromatic portion of Tyr109 and the bicyclic core of the ligand was also found. An additional hydrogen bond was made between the amide NH of the ligand and the backbone carbonyl oxygen atom of Thr49.

## 3. Discussion

The persistent circulation of the SARS-CoV-2 virus among the human population since its emergence in 2019 offers ample opportunities for the evolution of the virus in response to the selective pressure of the host immune system [29]. Currently circulating and novel viral variants show accumulation of mutations in critical residues of the spike protein conferring an increased ability to escape the host immune response deriving both from natural infection and vaccination. These limitations have prompted the development of next-generation vaccine strategies incorporating additional conserved viral antigens, including the Np protein [30,31]. Additionally, alternative antiviral approaches, such as siRNA-based therapeutics targeting SARS-CoV-2 replication, have shown promising antiviral activity [32,33]. Despite these advances, effective viral replication inhibitors remain critically important [34]. Remdesivir and Paxlovid (nirmatrelvir) are the main small-molecule antivirals approved for the treatment of COVID-19. Their molecular targets are the viral RNA-dependent RNA polymerase and the viral 3CL protease, respectively, both of which are classic targets in antiviral therapy. In other viral infections, pharmacological selective pressure has led to the emergence of resistance mutations against these inhibitor classes, a risk that also applies to SARS-CoV-2. Therefore, the development of new antivirals targeting different viral or host pathways is essential [35].

SARS-CoV-2 Np is a highly conserved protein across the Betacoronavirus family, showing a very low mutation rate [9]. In addition, it plays essential roles in the viral life cycle, contributing to viral gRNA duplication and packaging, as well as to the reorganization of the host intracellular environment to support viral replication [10]. Its modular architecture comprising NTD, CTD and IDRs, facilitates dynamic interactions with viral RNA and a range of host proteins. Recent studies have shown that Np undergoes RNA-driven liquid–liquid phase separation to form biomolecular condensates that enhance replication efficiency and protect viral RNA from host pattern recognition receptors [26,36].

In this work, we demonstrated that both the NTD and CTD of Np can bind dsRNA independently, with the CTD exhibiting higher intrinsic RNA-binding efficiency. Although the NTD is generally considered the primary RNA-binding region, several studies have reported that the CTD can also bind RNA, particularly structured or dsRNA [2,3,7,37]. Accordingly, the higher dsRNA-binding efficiency of the CTD observed in our assays likely reflects the nature of the RNA substrate. This finding supports a complementary role for the CTD in RNA recognition, particularly in the presence of host cofactors such as DDX3X. Indeed, the DEAD-box RNA helicase DDX3X enhances the binding affinity of both Np domains. This stimulation is mediated by direct protein–protein interaction, as demonstrated in pull-down assays using recombinant proteins. Notably, three truncated DDX3X constructs, DDX3X_(2–607)_, DDX3X_(132–607)_, and DDX3X_(132–662)_, were all capable of enhancing Np-RNA complex formation. DDX3X_(132–662)_, which includes the full RecA2 domain and its flanking residues, displayed the strongest physical interaction with Np, highly stimulating Np-RNA interaction. These findings suggest that DDX3X RecA2 domain constitutes the principal Np-binding interface.

AlphaFold 3 structural modelling provided a plausible 3D rationale for this interaction. The interaction map revealed that residues involved in DDX3X binding are clustered within the folded regions of the Np NTD and CTD. On the DDX3X side, the RecA2 domain contains several surface exposed residues that appear to contact Np, including segments adjacent to the helicase motifs. These predictions were validated by mammalian pull-down experiments using Halo-tagged RecA1 and RecA2 fragments. The interaction was strongest with the RecA2-containing fragment 414–662, confirming the predictive AlphaFold 3 model and pointing to RecA2 as a critical determinant of DDX3X interaction with Np. Together, these results support a model in which DDX3X, through its RecA2 domain, binds to both Np NTD and CTDs and potentially also to the Np disordered regions, enhancing its dsRNA binding activity. This cooperative interaction may increase the stability and efficiency of viral RNA packaging, promote RNP assembly and modulate the accessibility of RNA substrates for replication and translation.

Efficient interactions between Np domains and DDX3X variants were observed in mammalian cells, as confirmed by NanoBRET and Halo-TEV pull-down assays. These results support the physiological relevance of the interaction and indicate that it is not restricted to in vitro systems but also occurs in a cellular context. The AlphaFold 3 model also suggested the potential involvement of the IDRs in facilitating these interactions through conformational flexibility. This supports the confirmed function of these regions to promote LLPS, a process that is essential for RNP formation and efficient viral assembly [38]. Given that DDX3X is involved in stress granule dynamics and can undergo LLPS, co-condensation of DDX3X and Np may contribute to the spatial organization of replication–transcription complexes and to the disruption of host antiviral stress granule formation [12,39,40]. This mechanism reflects a broader viral strategy employed by other RNA viruses, including Influenza A and HCV, where host helicases are recruited to reorganize the intracellular RNA-protein network [41,42]. Thus, Np-DDX3X association may not only enhance viral replication but create a more permissive environment for viral propagation.

Furthermore, DDX3X plays a key role in innate immune signalling, particularly in activating the RIG-I-MAVS axis and type I interferon response [15]. Its sequestration or functional modulation by Np may therefore represent a strategic viral countermeasure. To directly interact with DDX3X, Np may interfere with its immune-stimulatory functions, thus delaying or reducing host antiviral responses. This is supported by previous findings that several RNA viruses, such as HIV-1 and HCV, also target DDX3X to suppress its antiviral functions and to promote viral replication [19,20,43]. Although this study focuses on DDX3X, Np may also participate in multivalent interactions with other helicases and RNA-binding proteins, forming higher-order complexes whose cooperative functions remain to be explored. In particular, DDX1, a close paralog of DDX3X, has been implicated in viral RNA sensing and replication and may play complementary or redundant roles in SARS-CoV-2 infection [10,11,22]. Future research into the interplay between Np and various helicases could reveal convergent strategies adopted by the virus to manipulate host RNA biology.

In addition, the broad interactome of Np highlights its potential as a vulnerable target for antiviral intervention, since disrupting these interactions could simultaneously impair multiple viral processes. Our screen of a small molecule collection found that compounds CT05 and CT10 reduce Np-dsRNA complex formation, suggesting that blocking the NTD-RNA binding groove could interfere with multiple stages of the viral life cycle. Given that this domain is conserved across coronaviruses, these compounds could exhibit a broad-spectrum of antiviral activity [9]. Furthermore, the disruption of the Np-DDX3X binding interface may synergistically inhibit viral replication by preventing both RNA binding and the functional sequestration of host helicase activity, offering a dual targeting therapeutic strategy.

## 4. Materials and Methods

### 4.1. Cloning, Expression and Purification of Recombinant Proteins

DDX3X and Np full-length proteins were cloned BamHI-NotI in pET30a(+) plasmid and expressed in BL21(DE3) *E. coli* cells with 0.5 mM isopropyl-β-D-1-thiogalacto-pyranoside (IPTG) (AppliChem, Darmstadt, Germany), overnight at 37 °C. Both proteins were purified by nickel affinity chromatography and imidazole gradient essentially as already described [11,44]. Then, both proteins were dialyzed, quantified and stored at −80 °C before use. DDX3X and Np truncated mutants were obtained by amplifying the wild type cDNAs with specific combined primers (Eurofins Genomics, Ebersberg, Germany) (Supplementary Table S1). For bacteria protein expression, DDX3X and Np mutants were cloned BamHI-NotI in pET-6HIS-TEV empty vector and purified with nickel affinity chromatography. Briefly, recombinant proteins were expressed in BL21(DE3) *E. coli* cells, DDX3X variants with 0.5 mM IPTG for 3 h at 37 °C, while Np truncated forms with 0.2 mM IPTG, overnight at 37 °C. Then, the bacterial cell pellets were lysed in lysis buffer (20 mM Tris pH 8, 200 mM KCl and protease inhibitors) and lysed by adding 1 mg/mL lysozyme, 10 µg/mL DNaseI and sonication. After centrifugation at 20,000 rpm for 1 h, the clear supernatants were incubated with Ni-NTA resin (Qiagen, Hilden, Germany), overnight at 4 °C. Finally, recombinant proteins were recovered using an 80–500 mM imidazole gradient. Fractions were analyzed in Coomassie Blue SDS-page gels (Supplementary Figure S7A–D) and purified fractions were pooled, dialyzed (20 mM Tris pH 8, 200 mM KCl, 10% glycerol) and stored at −80 °C. Concentration of purified proteins was calculated using ImageJ software version 1.54g and BSA protein as standard. For our purified proteins, the A260/A280 absorbance ratio was below 0.7 to exclude bacterial nucleic acid contamination. MBP-Np protein used in the bacteria pull-down experiment was obtained by subcloning BamHI-NotI the Np cDNA in the pET-6his-MBP-TEV expression vector. Instead, for expression in human U2OS cell line, DDX3X wild type and mutants were cloned EcoRV-NotI in pHTN-HaloTag (Promega, Madison, WI, USA), while Np full length, Np domains and Transglutaminase 2 (TGM2) gene were cloned KpnI-NotI in frame in pCMVTnT (Promega) vector containing standard NLuc reporter at the N-terminus [45]. The TGM2 gene was also subcloned EcoRI-NotI in the pHTN-HaloTag vector. Specific primers for cloning from the wt cDNAs are listed in Supplementary Table S1. The list of all the plasmids used in this work is in Supplementary Table S2.

### 4.2. Nucleic Acid Substrates

Nucleic acid substrates. RNA oligonucleotides were purchased from Biomers.net (Ulm, Germany). The sequences of the substrates used are:

ssRNA 38 mer
5′-AUGAAGGUUUGAGUUGAGUGGAGAUAGUGGAGGGUAGU-3′
ssRNA 18 mer FAM
3′-UACUUCCAAACUCAACUC-5′ FAM

For dsRNA, ssRNA oligonucleotides were mixed in a 1:1 (M/M) ratio in annealing buffer (30 mM HEPES–KOH pH 7.4, 100 mM KCl, 2 mM MgCl_2_, 50 mM NH_4_Ac) at a final concentration of 500 nM, heated at 95 °C for 5 min and then slowly cooled down at room temperature.

### 4.3. EMSA

EMSAs were performed by incubating 50 nM dsRNA 18/38 mer FAM with different concentrations of the purified proteins for 1 h at 4 °C in EMSA buffer (20 mM Tris-HCl pH 8, 2 mM DTT, 70 mM KCl, 2 mM MgCl_2_, 6U RNasin, 5% glycerol). When Np was used in the presence DDX3X, proteins were preincubated for 10 min at 4 °C before the addition of the dsRNA substrate. Reactions were stopped by adding 6× gel loading buffer (ThermoFisher Scientific, Waltham, MA, USA). Then, samples were separated by 12% TBE-polyacrylamide (19:1 SERVA Electrophoresis GmbH, Heidelberg, Germany) gel at 40 V for about 3 h in 1× TBE buffer at 4 °C in mini-PROTEAN electrophoresis system (Bio-Rad, Hercules, CA, USA). Fluorescent substrates and products were visualized at Typhoon-TRIO (GE Healthcare, Chicago, IL, USA). As a control experiment, we verified by nuclease assay that, under the same EMSA conditions, the purified proteins did not exhibit any residual RNase activity (Supplementary Figure S8). The intensity of the bands was measured by ImageJ densitometry to quantify the relative intensities of the overall shifted products with respect to the unbound substrate. Quantification was performed expressing the activity as relative shifted products, according to the formula S/(U + S) where U and S are the intensities of the Unbound and Shifted RNA molecules, respectively. Binding curves were generated by plotting the fraction of shifted RNA as a function of protein concentration and were fitted using GraphPad Prism 9.0 software. Data were analyzed using a one-site binding model under equilibrium conditions to estimate the apparent dissociation constant (K_d_) and the maximal shifted fraction (B_max_), which represents the plateau value at saturation rather than a kinetic parameter.

### 4.4. Pull-Down Assays

Bacteria pull-down assay was essentially performed as already described [11]. Briefly, MBP-Np protein was expressed in BL21(DE3) *E. coli* cells resuspended in 50 mM Tris pH 8, 150 mM NaCl, 0.05% NP40 (buffer A) and lysed by adding 10 mg/mL lysozyme, 1 mM EDTA and protease inhibitors. After sonication, the clear cell lysate corresponding to 10 µg of MBP-Np protein was bound to MBP resin Amintra (Abcam, Cambridge, UK) in the presence of 20 μg/mL of DNaseI and incubated for 2 h at 4 °C at 850 rpm on Thermomixer HC (Starlab srl, Milan, Italy). Then, after extensive washing with PBS 1×, 10 µg of purified DDX3X proteins were diluted to 150 µL in buffer A, added to the resin and incubated for 2 h at 25 °C on Thermomixer HC. The supernatant was discarded and the resin washed with PBS 1× and treated with 5U of TEV protease (Promega) in 150 µL of buffer A plus 1 mM DTT for 2 h at 25 °C, while stirring. Finally, the supernatant was recovered, and the collected samples were analyzed in Western blot. Control experiments were carried out by using BL21 (DE3) *E. coli* cell lysates transformed with pET 6HIS-MBP-TEV empty vector (#29656 Addgene, Watermort, MA, USA). Instead, cell pull-down assay was carried out using the HaloTag mammalian pull-down system (Promega) following manufacturer instructions with few modifications. Then, 0.5 × 10^6^ human U20S cells were seeded in six multiwell plates and transfected the next day using lipofectamine 2000 (ThermoFisher Scientific) with 3 µg of plasmid containing the wt or mutant versions of DDX3X and Np proteins. After 48 h, cells were lysed in 300 µL of PLB 1× buffer (Promega) containing protease inhibitors (Merck, Darmstadt, Germany) and centrifuged at 10,000 rpm for 10 min. Clear lysates were checked for transfection efficiency verifying NLuc expressed proteins with GloMax luminometer (Promega) and Halo expressed proteins with HaloTag TMR ligand. Quantification of expressed proteins was calculated by Western blot using purified proteins as standard. Then, pull-down assays were performed by mixing 3 µg of Halo tagged proteins with 3 µg of NLuc tagged proteins and 50 µL of pre-equilibrated HaloLink resin (Promega) to reach a total volume of 500 µL in TBST 1× with the addition of 20 μg/mL DNaseI and of 100 μg/mL RNase. Samples were incubated for 2 h under stirring at 25 °C and the HaloLink resin washed four times with TBS 1× and then incubated with 50 µL of TEV buffer 1× + 1 mM DTT + 2 µL of TEV plus protease (Promega) for 1 h while stirring at 25 °C. Finally, luminescence was calculated in sample supernatants using the Nano-Glo Luciferase assay system and Glomax luminometer (Promega).

### 4.5. Western Blot

Western blot analysis was performed according to standard procedures. Protein samples were separated by 10% SDS-PAGE, blotted onto Protran nitrocellulose membranes (GE Healthcare) and incubated overnight with mouse monoclonal anti-SARS-CoV-2 Np (1:1000) (SinoBiological, Beijing, China) and polyclonal rabbit anti-DDX3X A300-475A (1:1000) (Bethyl, Montgomery, TX, USA), followed by 1 h incubation with HRP-conjugated secondary antibodies goat anti-mouse or goat anti-rabbit (1:5000) (Merck). Chemiluminescent signals were developed using the ECL reagent and Chemidoc imaging system (Bio-Rad).

### 4.6. NanoBRET Assay

U2OS cells (ATCC HTB-96) were cultured in high-glucose Dulbecco’s Modified Eagle Medium (DMEM), supplemented with 10% fetal bovine serum (FBS) (Euroclone, Milan, Italy), and incubated at 37 °C with 5% CO_2_. Cells were seeded one day prior to transfection. The NanoBRET assay was conducted based on the guidelines provided in the NanoBRET protein/protein interaction system technical manual (Promega). U2OS cells were seeded in 6-well plates and co-transfected using lipofectamine 2000, maintaining a donor-to-acceptor plasmid ratio of 1:10 (200 ng NanoLuc construct and 2 µg HaloTag construct). After 48 h, cells were trypsinized and resuspended in phenol red-free Opti-MEM (ThermoFisher Scientific) containing 4% FBS, at a density of 2 × 10^5^ cells/mL. The suspension was mixed with HaloTag 618 ligand (1 µL/mL), and 100 µL per well was dispensed into a 96-well plate. Plates were incubated for 16 h at 37 °C prior NanoBRET measurement. To detect the BRET signal, Nano-Glo substrate was diluted in phenol red-free Opti-MEM at 10 µL/mL, and 25 µL of this solution was added to each well. Measurements were taken using the GloMax luminometer (Promega) with preconfigured filters for donor emission (450 nm/8 nm BP) and acceptor emission (600 nm LP). The preloaded NanoBRET 618 protocol was selected from the instrument menu. The raw NanoBRET ratio was calculated by dividing the acceptor emission 618 nm by the donor emission 460 nm for each sample. To convert these raw values to milliBRET units (mBU), each ratio was multiplied by 1000. NanoBRET plasmids for the expression of p53-HaloTag and NLuc-MDM2 positive controls were purchased from Promega. For the negative controls, NLuc-TGM2 plasmid was used in combination with Halo-DDX3X plasmid or Halo-TGM2 plasmid was used in combination with NLuc-Np full length plasmid.

### 4.7. Computational Details

The three-dimensional structural model of the DDX3X-Np complex was built using AlphaFold 3, which was specifically conceived to predict protein–protein and protein–ligand structures [46]. The amino acid sequences of DDX3X (UniProt ID: O00571) and Np (UniProt ID: A0A6B9VLF5) were manually loaded into AlphaFold server. To represent the RNA component, two complementary ssRNA sequences, corresponding to a 18/38 mer dsRNA used in our EMSA, were entered as separate chains without a preassembled 3D structure. An ATP molecule was also included as a ligand allowing AlphaFold to predict its binding site. The final model was examined with ChimeraX 1.9 using the analysis tools to exclude steric clashes and atomic overlaps and to identify the residues involved in the interactions between DDX3X and the Np. No significant steric or electrostatic violations were detected in the final model, supporting the structural feasibility of the predicted interactions. NCI, DrugBank, and MolPort databases of commercially available compounds were used for virtual screening procedures. NCI database contains about 280,000 compounds, which were available for free at the time of this study. DrugBank database contains more than 10,000 compounds that represent known drugs or compounds that accessed clinical trials (in particular, they are classified as approved, experimental, nutraceutical, illicit, withdrawn, and investigational), which can be used for drug repurposing studies. MolPort database contains more than 6 million compounds and represents one of the most comprehensive databases of small molecules. The bi-dimensional structures of the compounds belonging to NCI, DrugBank, and MolPort databases were prepared with the LigPrep routine within the Schrodinger suite [47] by generation of tautomeric and ionization states at pH 7.4 ± 0.5 using the Epik software version 5.5 and sampling ring conformations. Next, each of the three-dimensional ligand structures was submitted to a conformational analysis that generated 100 conformations, in turn minimized by using the OPLS3e force field. The resulting multi-conformer databases were pruned based on Lipinski’s rule of five and then used for docking-based virtual screening. Fifteen three-dimensional structures of Np-NTD RNA binding domain alone or in complex with RNA fragments were downloaded from the protein data bank (Supplementary Table S3). Among them, eleven structures have been determined by X-ray crystallography (namely, 6m3m, 6vyo, 6wkp, 7cdz, 7uw3, 7vbd, 7vnu, 7xwz, 7xx1, 8iqj, and 8x1h), while the remaining have been obtained by solution or solid phase NMR experiments (namely, 6yi3, 7acs, 7act, and 7sd4). Only three structures of them represented complexes between the NTD portion of the Np and RNA fragments (namely, 7acs, 7act, and 7xwz). Protein structures were prepared with the Protein Preparation Wizard of the Schrodinger suite, by assigning bond orders, adding hydrogen atoms, and capping terminal residues. A final restrained minimization was also performed on the protein structures until convergence (RMSD = 0.30 Å), by using the OPLS3e force field. In the next step, a grid was generated, which contains information on the size, shape, and physico-chemical properties of the putative binding site, required for an accurate scoring of the ligand poses during docking simulations. Hydroxyl groups of Tyr and Ser residues and thiol groups of Cys residues were allowed to rotate. Finally, all the conformations of each small molecule contained in the multi-conformer databases were iteratively docked into the protein binding site by using the software Glide version 9.0 with standard precision. The resulting complexes were submitted to a post-docking minimization with default parameters, thus leading to a list of putative hit compounds that were prioritized based on their Glide score. Ten compounds (98% purity as declared by the vendors) were purchased from MolPort and submitted to biological evaluation.

## 5. Conclusions

This study uncovers an important functional interaction between the SARS-CoV-2 Np and the host DEAD-box helicase DDX3X, shading new light on the molecular process used by SARS-CoV-2 to subvert host cellular machinery to support viral replication. We showed that DDX3X directly interacts with structured domains of Np and enhances its ability to bind dsRNA, suggesting a synergistic mechanism to facilitate viral RNA packaging and replication–transcription complex formation. This interaction could also interfere with the host antiviral defence because DDX3X is known to participate in immune signalling, RNA metabolism, and stress granule formation. While our conclusions are derived from experiments using short dsRNA substrate, future studies employing longer viral RNA fragments or full-length genomic RNA will be required to fully establish the role of this interaction during SARS-CoV-2 infection. Structural modelling and experimental validation suggest that DDX3X RecA2 domain act as the principal interface for Np binding, indicating a specific targetable interaction surface. Of particular interest, the discovery of small molecule inhibitors that reduce Np-dsRNA binding identifies a promising antiviral strategy, especially against conserved domains with broad spectrum activity potential. Moreover, interfering with the Np-DDX3X interface may offer a dual mechanism of action blocking both RNA binding and the recruitment of host factors. These findings offer new opportunities for the design of broad-spectrum therapeutics targeting multifunctional viral–host interfaces critical to the coronavirus life cycle.

## Figures and Tables

**Table 1:** Effects of DDX3X and Np combinations on dsRNA binding efficiency.

Protein	K_d_ (nM)	B_max_/K_d_ (pmol·µM^−1^)
Np CTD_(247–364)_	91.9 ± 9.5	3.1 ± 0.5
Np CTD_(247–364)_ + DDX3X	55.9 ± 6.5	7.5 ± 0.8
Np NTD_(41–186)_	94.3 ± 7.0	2.7 ± 0.3
Np NTD_(41–186)_ + DDX3X	59.3 ± 9.8	4.9 ± 0.6
Np NTD_(41–212)_	88.6 ± 6.3	2.7 ± 0.5
Np NTD_(41–212)_ + DDX3X	66.6 ± 2.8	4.4 ± 0.6
DDX3X_(2–607)_ + Np	27.2 ± 3.7	15.1 ± 0.9
DDX3X_(132–607)_ + Np	25.2 ± 2.5	17.4 ± 1.1
DDX3X_(132–662)_ + Np	20.5 ± 3.4	20.5 ± 0.6

## Data Availability

The original contributions presented in this study are included in the article/Supplementary Material. Further inquiries can be directed to the corresponding author.

## Supplementary Materials

The following supporting information can be downloaded at: https://www.mdpi.com/article/10.3390/ijms27020672/s1.

## Abbreviations

The following abbreviations are used in this manuscript:

Np	SARS-CoV-2 nucleocapsid protein
RNP	Ribonucleoprotein complex
gRNA	Genomic RNA
LLPS	Liquid–liquid phase separation
NTD	N-terminal domain
CTD	C-terminal domain
IDRs	Intrinsically disordered regions
LKR	Central linker region
dsRNA	Double stranded RNA
RIG-I	Retinoic acid-inducible gene-I
IPS-1	Interferon-beta promoter stimulator 1
HCV	Hepatitis C virus
EMSA	Electrophoretic Mobility Shift Assay
MBP	Maltose binding protein
NLuc	NanoLuc luciferase
TGM2	Transglutaminase 2
pTM	Predicted Template Modelling score
ipTM	Interface predicted TM-score
pLDDT	Predicted local distance difference test
RMSD	Root Mean Square Deviation
