# Supplementary material for: Dissecting the Interaction Domains of SARS-CoV-2 Nucleocapsid Protein and Human RNA Helicase DDX3X and Search for Potential Inhibitors
Source: Int J Mol Sci. 2026 Jan 9;27(2):672. doi: 10.3390/ijms27020672 (PMC12841228; doi:10.3390/ijms27020672)
Supplement: Supplementary file 1 [file ijms-27-00672-s001.zip › ijms-4010523-supplementary.pdf]

Primer name	DNA sequence
Np CTD aa247 BamHI For	CGCGGATCCACCAAGAAGTCTGCTGCTGAGGCA
Np CTD stop aa364 NotI Rev	TAGCGGCCGCCTAAGGAAAGGTCTTGTAGGCATCA
Np NTD aa41 BamHI For	CGCGGATCCAGACCACAGGGACTGCCAAACAAC
Np NTD stop aa186 NotI Rev	ATAGCGGCCGCCTAGGACCTGCTGGATGCCTGGCT
Np NTD stop aa212 NotI Rev	ATAGCGGCCGCTCAGCCAGCCATCCTGGCAGGGCT
DDX3X aa2 BamHI For	CATGGATCCAGTCATGTGGCAGTGGAAAATGC
DDX3X stop aa662 NotI Rev	ATATAGCGGCCGCTCAGTTACCCCACCAGTCAACC
DDX3X aa132 BamHI For	CGCGGATCCGATGAAGATGATTGGTCAAAACCA
DDX3X aa607 stop NotI Rev	AAGCGGCCGCCTAACCGCTACTTTGTCGGTAGTCT
Np NTD aa41 KpnI For	CGGGGTACCATGAGACCACAGGGACTGCCAAAC
Np CTD aa247 KpnI For	CGGGGTACCATGACCAAGAAGTCTGCTGCTGAGG
DDX3X EcoRV For	GCTAGGATATCAATGAGTCATGTGGCAGTGGAAAA
DDX3X aa132 EcoRV For	GCTAGGATATCAGATGAAGATGATTGGTCAAAACC
DDX3X aa182 EcoRV For	GCTAGGATATCATTCAGTGATGTTGAGATGGGAGA
DDX3X aa404 stop NotI Rev	AAGCGGCCGCTTAAGCCAAGAAGATATATTCATCTAAG
DDX3X aa414 EcoRV For	CTAGGATATCAAACATCACACAGAAAGTAGTTTGG
DDX3X aa544 stop NotI Rev	AAGCGGCCGCTTAGAATGAGGTTGCCAGGCCAAGG
TGM2 KpnI For	GATGGTACCGCCGAGGAGCTGGTCTTAGAGAGG
TGM2 stop NotI Rev	ATTGCGGCCGCCTAGTCGACTCTAGAGGTACCG

Supplementary Table ST1. Specific primers for the amplification of Np and DDX3X domains.

Plasmid name
pET30a(+)-DDX3X full length aa 2-662
pET-6HIS-DDX3X aa 2-607
pET-6HIS-DDX3X aa 132-607
pET-6HIS-DDX3X aa 132-662
pET30a(+)-Np full length aa 2-419
pET-6HIS-MBP-TEV-Np full length
pET-6HIS-TEV- Np NTD aa 41-186
pET-6HIS-TEV- Np NTD aa 41-212
pET-6HIS-TEV- Np CTD aa 247-364
pHTN-Halotag-TEV-DDX3X full length aa 2-662
pHTN-Halotag-TEV-DDX3X aa 2-607
pHTN-Halotag-TEV-DDX3X aa 132-607
pHTN-Halotag-TEV-DDX3X aa 132-662
pHTN-Halotag-TEV-TGM2
pCMVTnT-NLuc-TGM2
pCMVTnT-NLuc-Np full length aa 2-419
pCMVTnT-NLuc-Np NTD aa 41-186
pCMVTnT-NLuc-Np Np NTD aa 41-212
pCMVTnT-NLuc-Np CTD aa 247-364
pHTN-Halotag-TEV-DDX3X RecA1 aa 182-404
pHTN-Halotag-TEV-DDX3X RecA2 aa 414-544
pHTN-Halotag-TEV-DDX3X RecA2 aa 414-607
pHTN-Halotag-TEV-DDX3X RecA2 aa 414-662

Supplementary Table ST2. List of all the plasmids used.

PDB ID	Method[1]	Complexed ligand
6m3m	X-ray (2.70)	
6vyo	X-ray (1.70)	
6wkp	X-ray (2.67)	
6yi3	Solution NMR	
7acs	Solution NMR	7mer dsRNA
7act	Solution NMR	10mer ssRNA
7cdz	X-ray (1.80)	
7sd4	Solid-state NMR	
7uw3	X-ray (1.70)	
7vbd	X-ray (1.94)	
7vnu	X-ray (1.95)	
7xwz	X-ray (2.25)	6mer dsRNA
7xx1	X-ray (1.90)	
8iqj	X-ray (2.30)	
8x1h	X-ray (2.00)	

Supplementary Table ST3. List of three-dimensional structures of SARS-CoV-2 Np NTD-RNA binding domain. [1]In parentheses, resolution in Å.

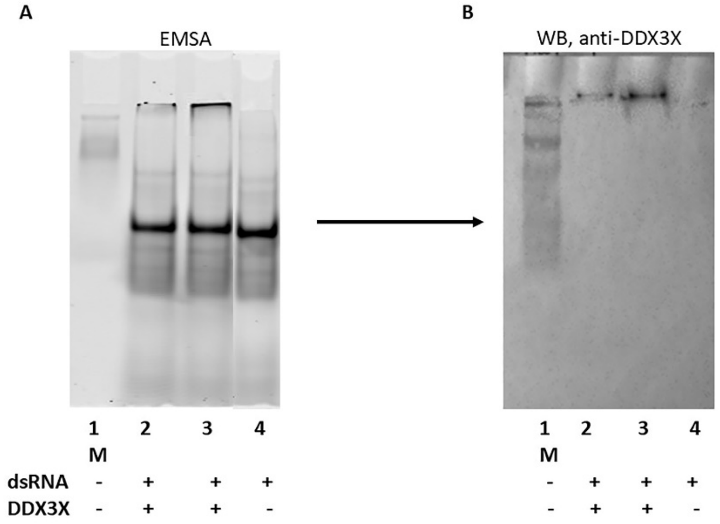

Supplementary Figure S1. WEMSA assay. (**A**) Typhoon fluorescence visualization of the dsRNA 18/38 mer. (**B**) Western blot of the same gel after nitrocellulose transfer. In both panels dsRNA and DDX3X are indicated when added. Lane 1, W.B. molecular weight marke; Lane 2, DDX3X is 0.25 μM; Lane 3, DDX3X is 0.5 μM.

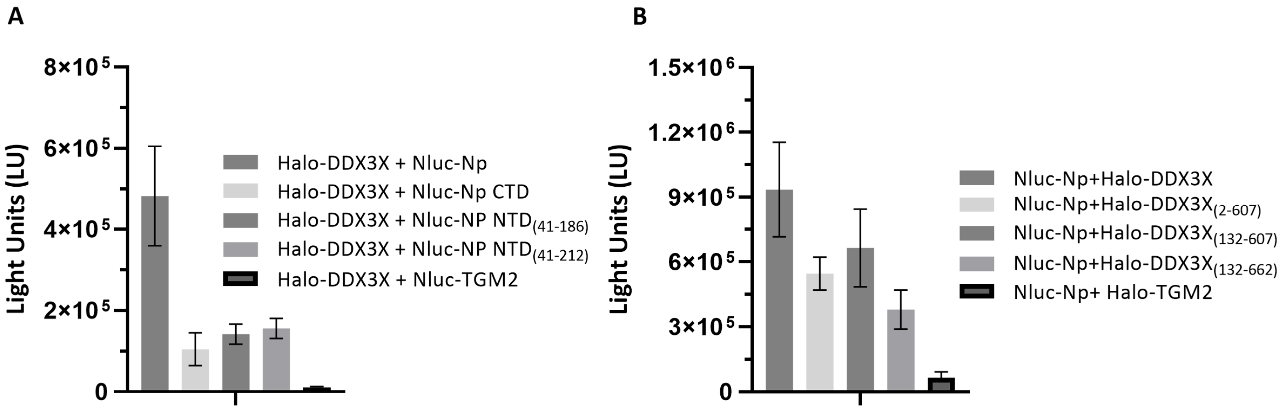

Supplementary Figure S2. Halo-TEV pull-down system. (**A**) Pull-down of Halo-DDX3X full length protein with Np full length or NTD and CTD domains tagged with NanoLuc. (**B**) Pull-down of NanoLuc-Np full length with DDX3X full length or truncated forms tagged with Halo. In both panels the negative controls with NLuc-TGM2 or Halo-TGM2 were included as in indicated in material and methods. Values expressed in light units (LU) are the means ± SD of three independent experiments.

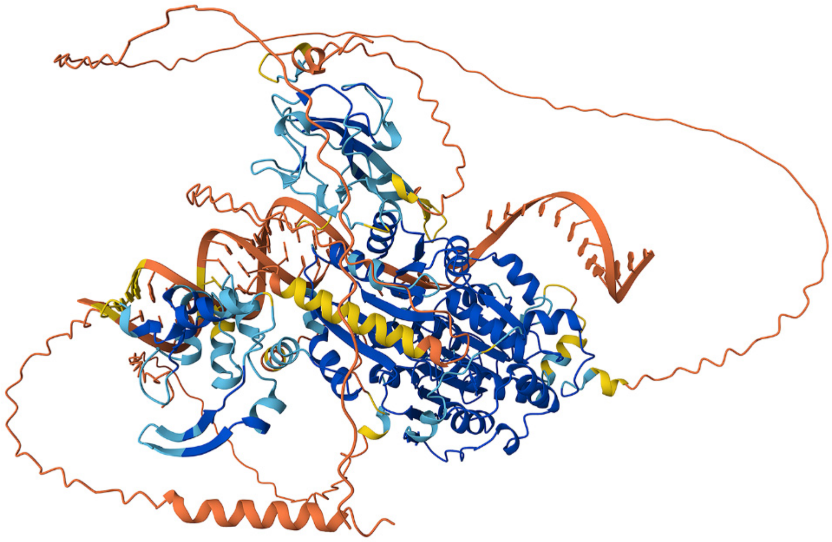

Supplementary Figure S3. Cartoon representation of the complex between DDX3X, Np, dsRNA18/38mer, and ATP molecule, as modelled by AlphaFold 3 (ipTM = 0.49, pTM = 0.52).

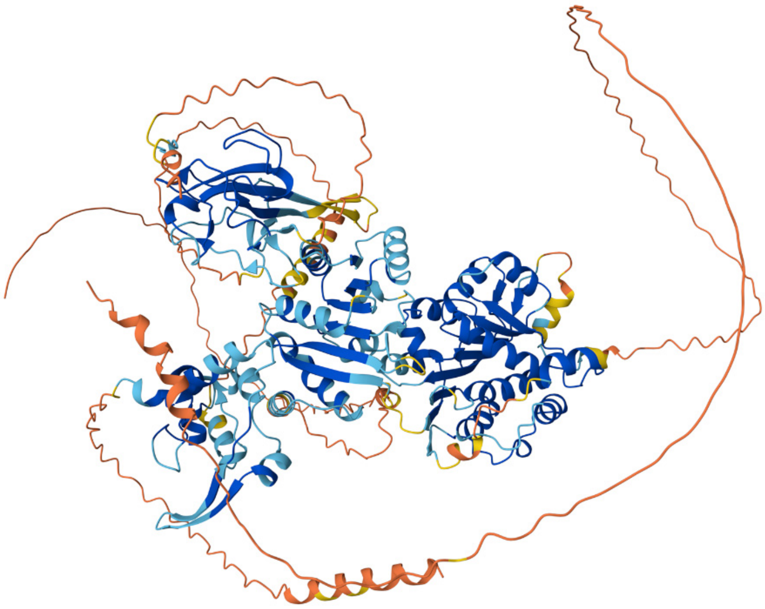

Supplementary Figure S4. Cartoon representation of the complex between DDX3X and Np alone, as modelled by AlphaFold 3 (ipTM = 0.24, pTM = 0.38).

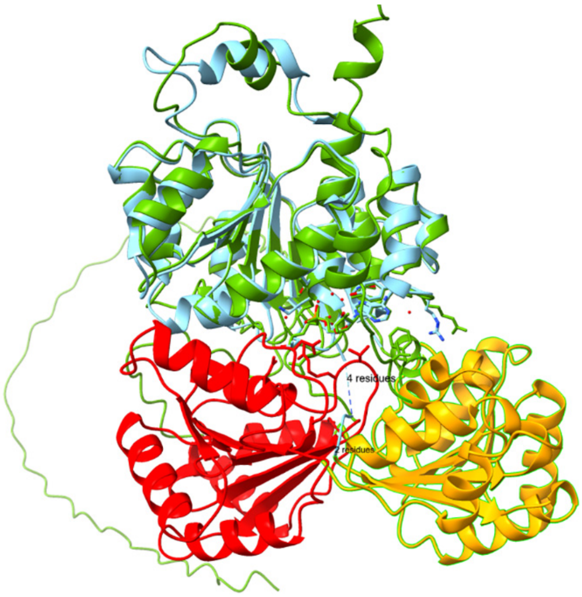

Supplementary Figure S5. Cartoon representation of DDX3X alignment. AlphaFold 3 DDX3X structure is coloured in green (RecA1 domain) and red (RecA2 domain), while DDX3X from PDB ID 2I4I crystal structure is coloured in light blue (RecA1 domain) and yellow (RecA2 domain). The crystal structure of DDX3X consists of only 417 amino acids (aa 168-582) obtained without the disordered N and C terminal regions that were instead included in our AlphaFold model. The two structures overlay with a RMSD value of 0.736 Å. RecA2 domains are rotated of 175.66 degrees with a translation of 28.24 Å.

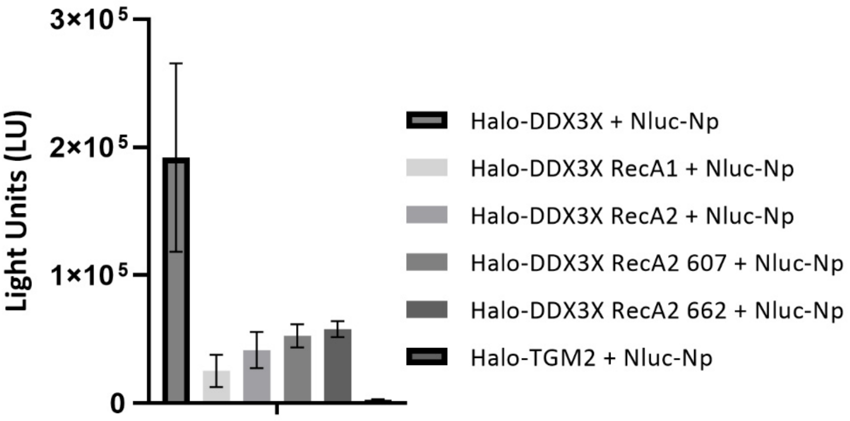

Supplementary Figure S6. Halo-TEV pull-down system for DDX3X domains and Np interaction. Pull-down of Halo-DDX3X full length protein and Halo-DDX3X RecA1 and RecA2 domains with Np full length. Values expressed in light units (LU) are the means ± SD of two independent experiments.

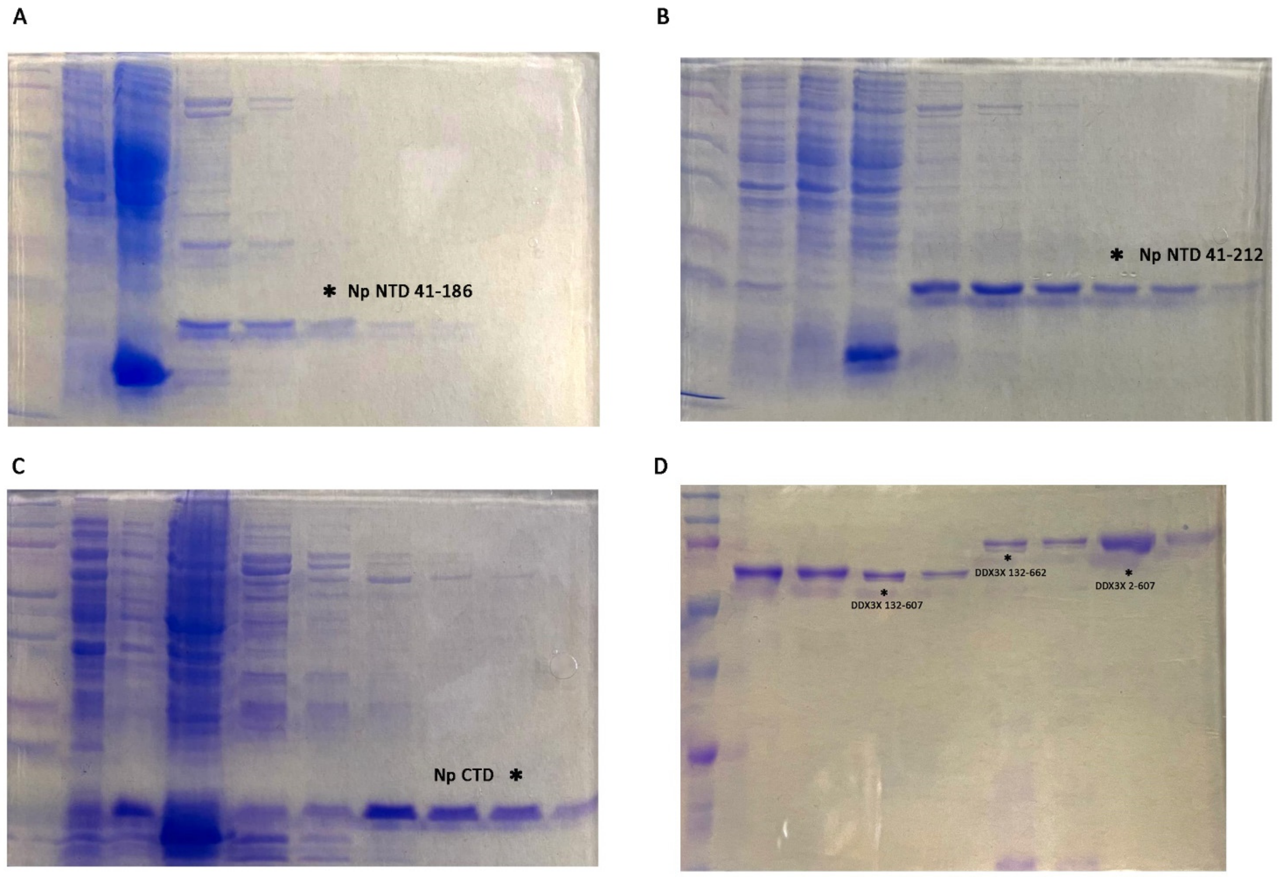

Supplementary Figure S7. Coomassie of purified proteins after TEV treatment. (**A**) Np NTD$_{(41-186)}$. (**B**) Np NTD$_{(41-212)}$. (**C**) Np CTD$_{(247-364)}$. (**D**) DDX3X $_{(132-607)}$, DDX3X $_{(132-662)}$. and DDX3X $_{(2-607)}$. *In all the panels indicates the purified fraction used in the experiments.

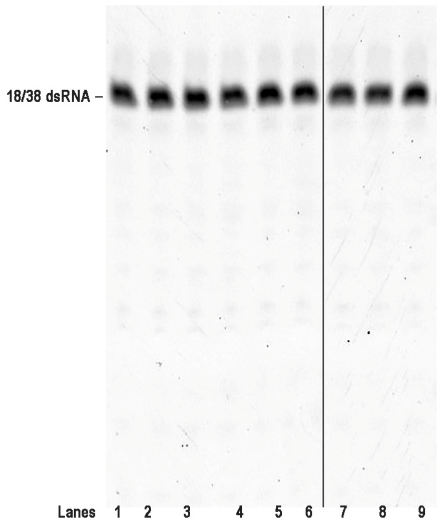

Supplementary Figure S8. Nuclease assay of FAM labelled 18/38 mer dsRNA after 1 h incubation at 4 °C with purified proteins. Samples were run on a 7 M urea - 12% polyacrylamide (19:1) denaturing gel. Lane 1, FAM 18/38 mer dsRNA not treated, Lane 2, treated with 0.5 μM DDX3X; Lane 3, treated with 0.5 μM $DDX3X_{(2-607)}$; Lane 4, treated with 0.5 μM $DDX3X_{(132-607)}$; Lane 5, treated with 0.5 μM $DDX3X_{(132-662)}$; Lane 6, treated with 0.5 μM Np, Lane 7, treated with 0.5 μM Np $NTD_{(41-186)}$; Lane 8, treated with 0.5 μM Np $NTD_{(41-212)}$; Lane 9, treated with 0.5 μM Np $CTD_{(247-364)}$.
