# Supplementary figures and images for: Dissecting the Interaction Domains of SARS-CoV-2 Nucleocapsid Protein and Human RNA Helicase DDX3X and Search for Potential Inhibitors
Source: Int J Mol Sci. 2026 Jan 9;27(2):672. doi: 10.3390/ijms27020672 (PMC12841228; doi:10.3390/ijms27020672)

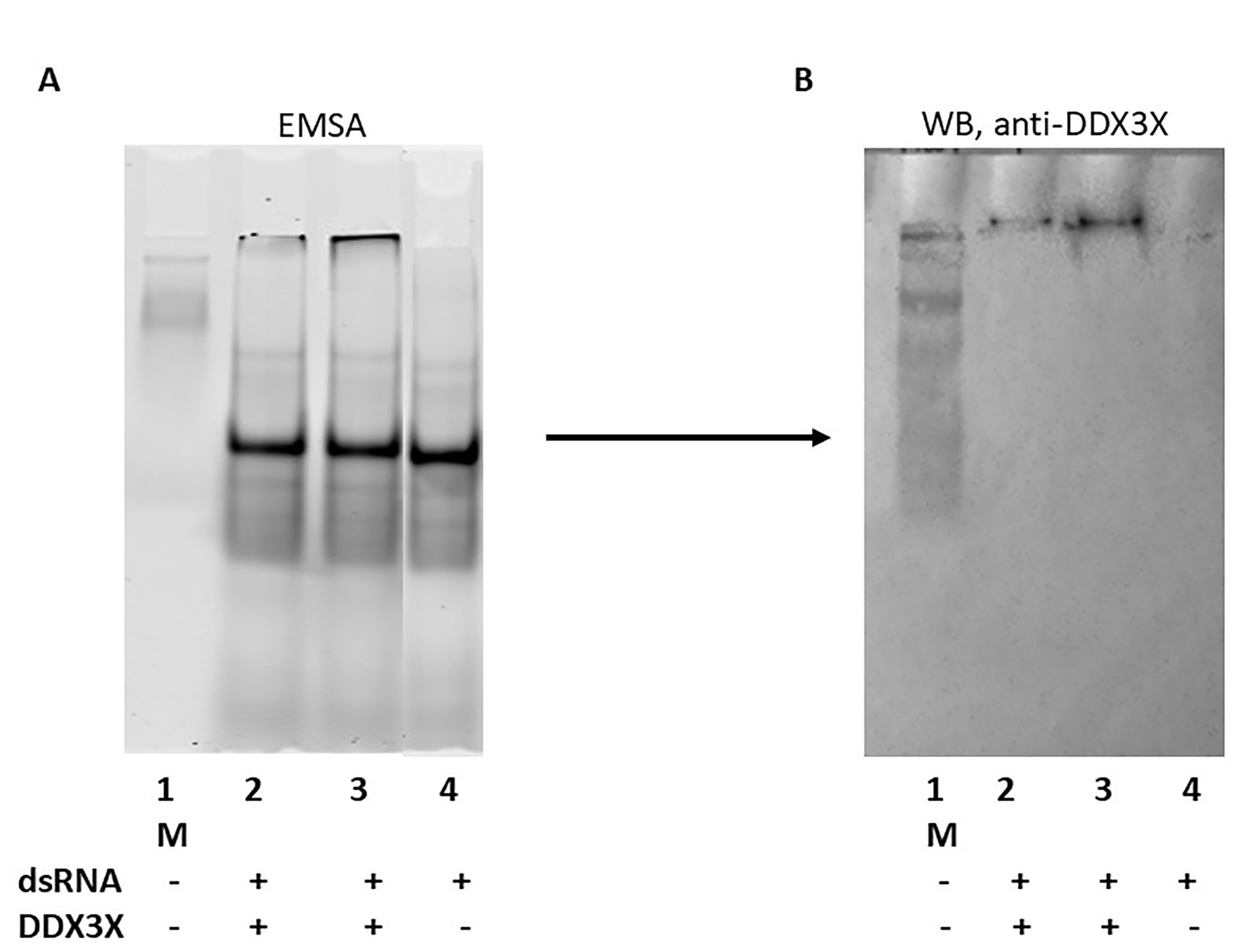

Supplement: Supplementary file 1 [file ijms-27-00672-s001.zip › Figure S1.tif]

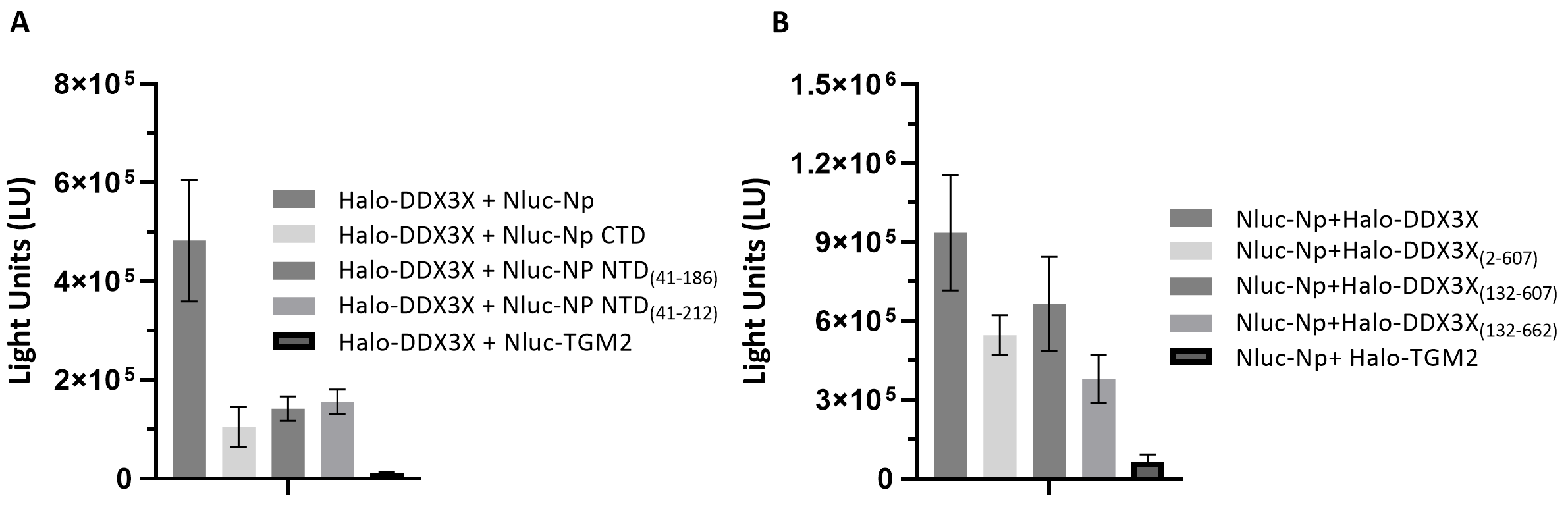

Supplement: Supplementary file 1 [file ijms-27-00672-s001.zip › Figure S2.tif]

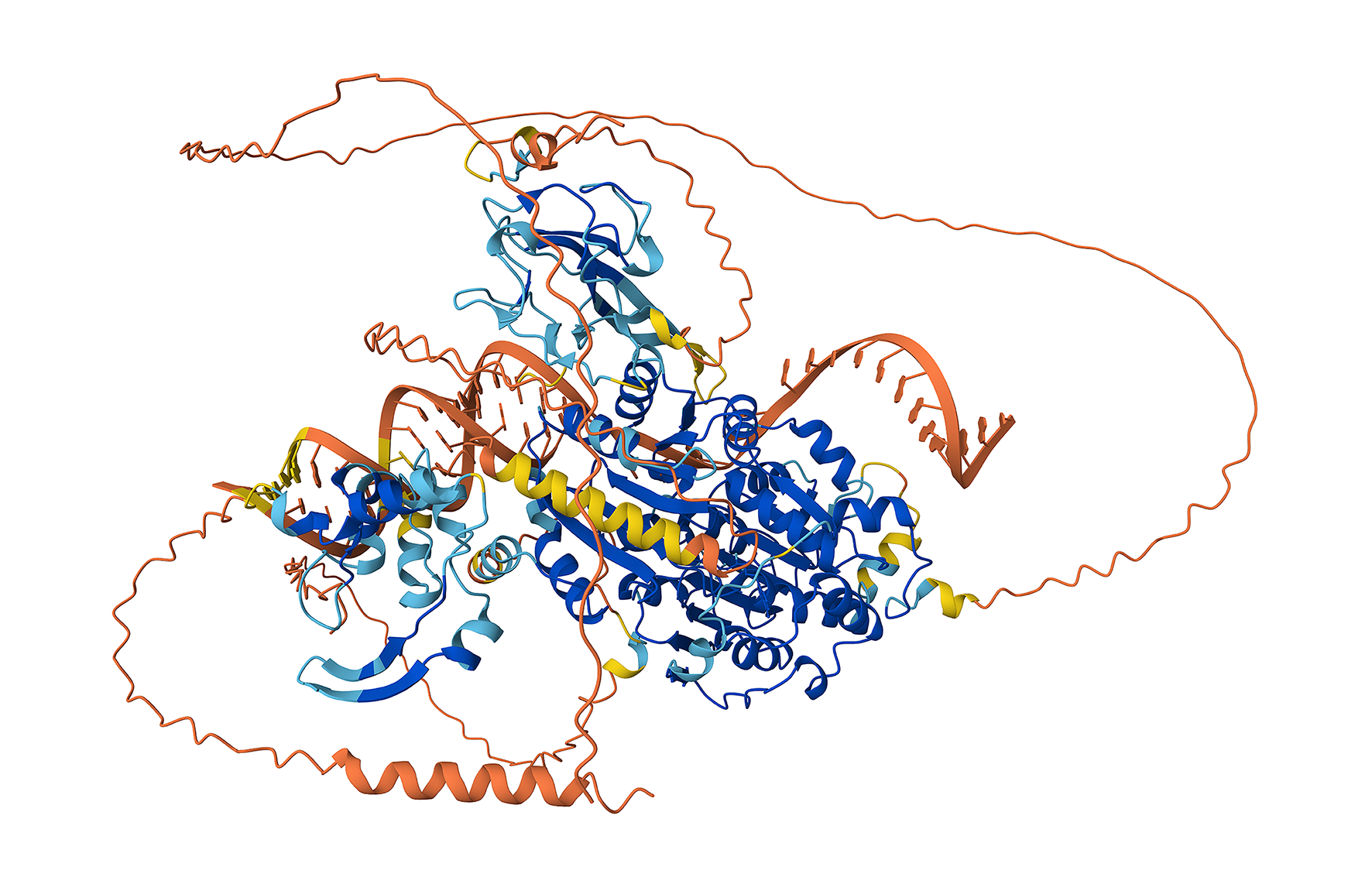

Supplement: Supplementary file 1 [file ijms-27-00672-s001.zip › Figure S3.tif]

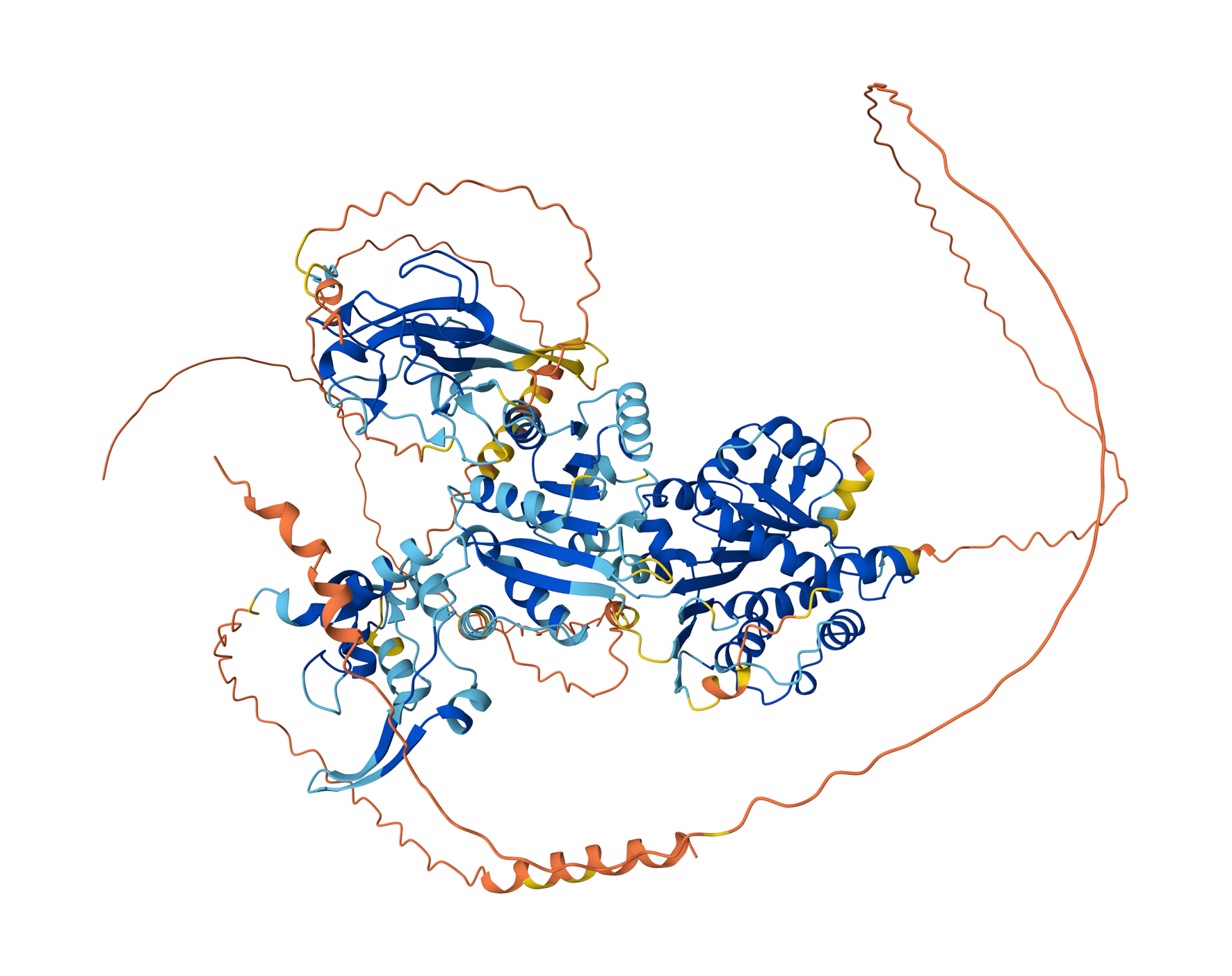

Supplement: Supplementary file 1 [file ijms-27-00672-s001.zip › Figure S4.tif]

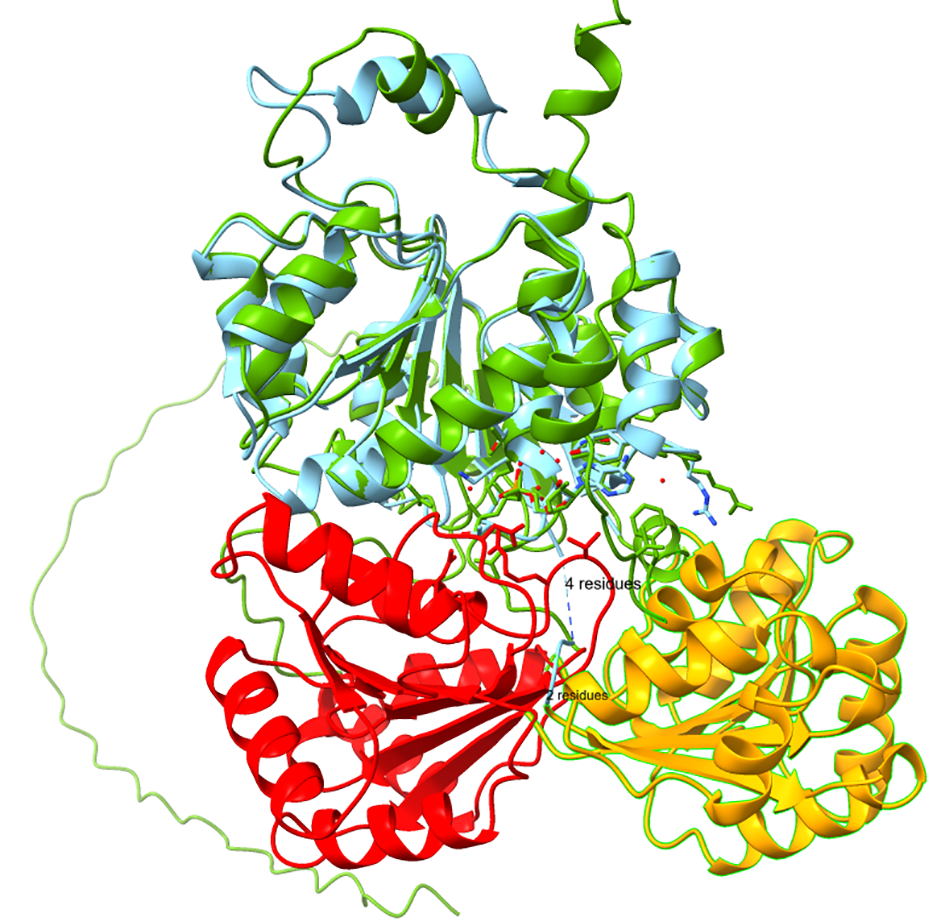

Supplement: Supplementary file 1 [file ijms-27-00672-s001.zip › Figure S5.tif]

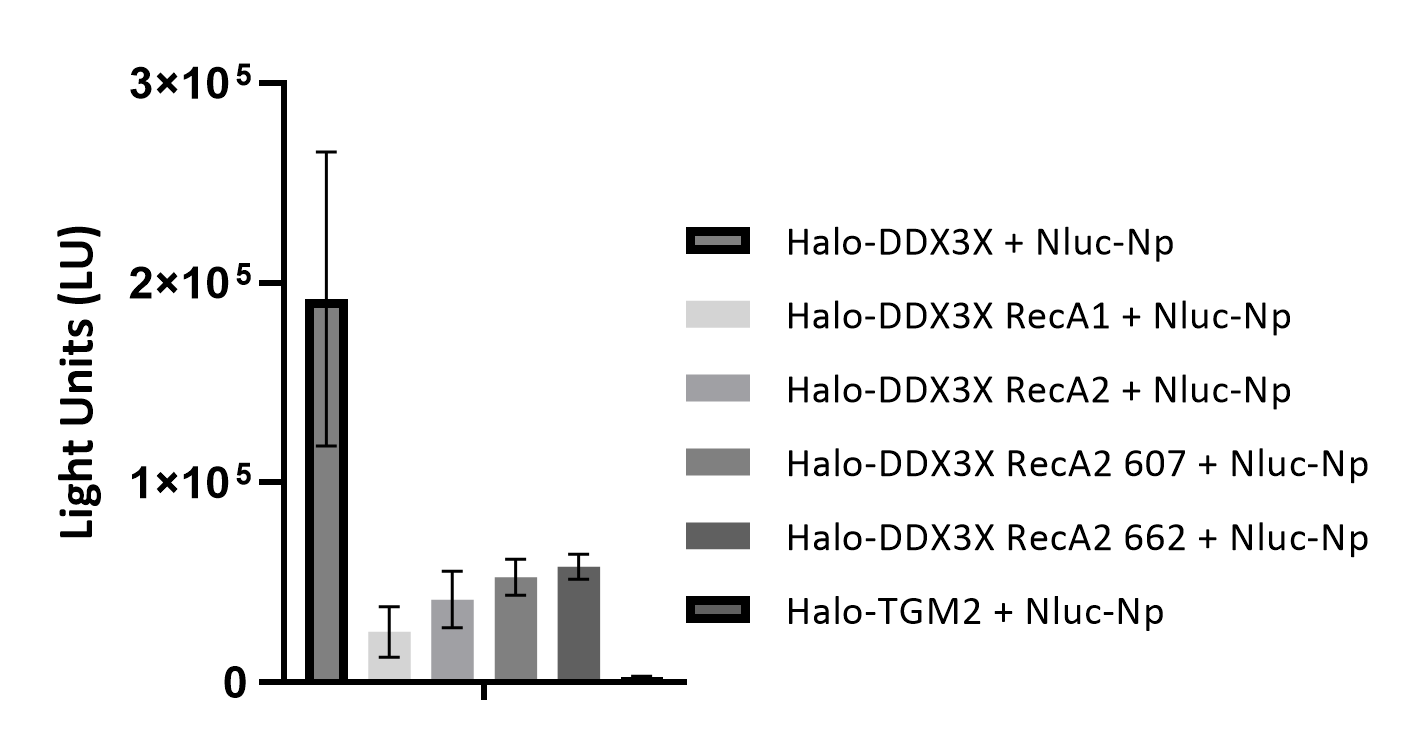

Supplement: Supplementary file 1 [file ijms-27-00672-s001.zip › Figure S6.tif]

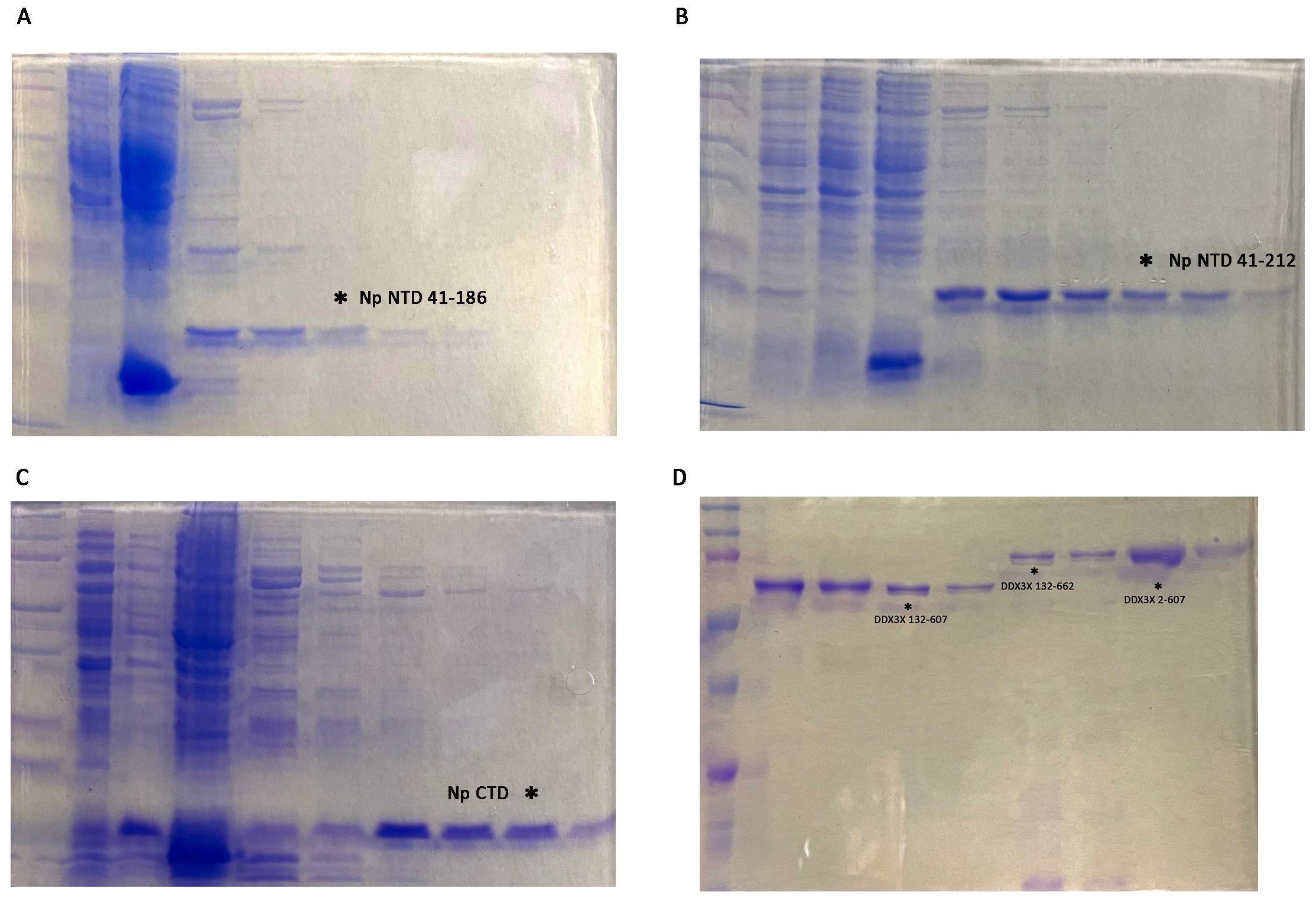

Supplement: Supplementary file 1 [file ijms-27-00672-s001.zip › Figure S7.tif]
